# Gut microbiota regulates chronic ethanol exposure-induced depressive-like behavior through hippocampal NLRP3-mediated neuroinflammation

**DOI:** 10.1038/s41380-022-01841-y

**Published:** 2022-10-24

**Authors:** Hui Yao, Dalin Zhang, Hao Yu, Huiya Yuan, Hui Shen, Xinze Lan, Hao Liu, Xiaohuan Chen, Fanyue Meng, Xu Wu, Guohua Zhang, Xiaolong Wang

**Affiliations:** 1 Department of Forensic Pathology, China Medical University School of Forensic Medicine, Shenyang, 110122 Liaoning PR China; 2 Liaoning Province Key Laboratory of Forensic Bio-evidence Sciences, Shenyang, 110122 Liaoning PR China; 3 China Medical University Center of Forensic Investigation, Shenyang, 110122 Liaoning PR China; 4 Department of Thyroid Surgery, The First Affiliated Hospital of China Medical University, Shenyang, 110001 Liaoning PR China; 5 Department of Forensic Analytical Toxicology, China Medical University School of Forensic Medicine, Shenyang, 110122 Liaoning PR China

**Keywords:** Physiology, Neuroscience, Psychology

## Abstract

Chronic ethanol exposure (CEE), which can lead to neuroinflammation, is an increasing risk factor for depression disorder, but the underlying mechanism is not clear. Recent observations have revealed the associations among psychiatric disorders, ethanol exposure and alterations of the gut microbiota. Here, we found that CEE induced depressive-like behavior, which could be alleviated by probiotics and transferred from donor to recipient mice by fecal microbiota transplantation (FMT). Neuroinflammation and the activation of the NLRP3 inflammasome were also observed in recipient mice. The downregulation of NLRP3 in the hippocampus mitigated CEE-induced depressive-like behavior and neuroinflammation but had no significant effect on FMT recipient mice. Moreover, elevated serum inflammatory factors in recipient mice showed a significant mediation effect between the gut microbiota and depressive-like behavior. Together, our study findings indicate that the gut microbiota contributes to both hippocampal NLRP3-mediated neuroinflammation and depressive-like behavior induced by CEE, which may open avenues for potential interventions against CEE-associated psychiatric disorders.

## Introduction

Ethanol, also known as alcohol, is one of the most commonly abused substances in the world [1]. Ethanol freely crosses the blood–brain barrier and is particularly toxic to the brain. The toxic effects of ethanol on the brain manifest in many aspects, including the impairment of memory and learning [2–4]. Notably, epidemiological studies have shown that ethanol exposure increases the risk of major depressive disorder (MDD) [5–7]. Animal experiments also demonstrated that ethanol is an important risk factor for mental disorders such as anxiety and depression [8–10]. However, the mechanisms involved are still not completely understood.

In recent years, the role of the gut microbiota has been increasingly studied. The gut microbiota can affect the central nervous system through a variety of pathways and participate in the pathogenesis of neuropsychiatric disorders [11–13]. Previous studies have noted that ethanol affects gastrointestinal homeostasis and the composition of the gut microbiota, but these studies have mostly focused on the digestive system. With the rise of the microbiota-gut-brain axis theory, the association between the gut microbiota and the neurotoxic effects of ethanol has attracted much attention [9, 14, 15].

As a psychiatric disorder with a complex etiology, depression is affected not only by genetic or environmental factors but also by immune activation and the release of proinflammatory cytokines. Clinical studies have observed increased inflammatory cytokines or neuroinflammation in patients with MDD [16, 17]. Inflammasomes are an important part of the innate immune system that can recognize exogenous and endogenous danger signals, and the NLRP3 inflammasome is the most representative inflammasome [18, 19]. NLRP3-mediated neuroinflammation and the activation of microglia are important causes of exacerbating depressive symptoms [20]. Clearing the intestinal flora of mice with broad-spectrum antibiotics mitigated the activation of the NLRP3 inflammasome and neuroinflammation in the hippocampus induced by ethanol exposure [21]. This finding suggests that changes in intestinal flora prior to ethanol exposure are likely dependent on the activation of the NLRP3 inflammasome.

To explore the role of the gut microbiota and the underlying mechanism in chronic ethanol exposure (CEE)-induced depressive-like behavior, we used a 90-day CEE mouse model and added probiotic administration as a comparison. Hippocampal NLRP3-downregulated mice were obtained by adeno-associated virus (AAV) microinjection, and a CEE model was also established. We also used fecal microbiota transfer (FMT) to eliminate the direct effects of ethanol. Changes in behavior and emotion, the gut microbiota, serum cytokines, hippocampal neuronal damage and neuroinflammation were detected. We also introduced causal mediation analysis to explore the associations among the gut microbiota, inflammatory cytokines and depressive-like behavior. Our findings suggest that the gut microbiota regulates CEE-induced depressive-like behavior through hippocampal NLRP3-mediated neuroinflammation and that serum inflammatory cytokines may mediate the association between the gut microbiota and depressive-like behavior in CEE-exposed mice.

## Methods

### Animals and chemicals

Male C57BL/6N mice were purchased from the Laboratory Animal Center of China Medical University. Mice were housed two animals per cage with free access to water and food. The indoor temperature was controlled at 21 ± 1 °C, the relative humidity was 50% ± 10%, and the light cycle was 12 h (6:00–18:00). All animal procedures were conducted in accordance with the Guidelines for the Care and Use of Laboratory Animals of China Medical University (approval code: CMU2020103). All experimental procedures were performed according to the National Institutes of Health Guide for the Care and Use of Laboratory Animals (NIH Publications No. 8023, revised 1978).

All chemicals used in this study were of analytical grade. Unless stated otherwise, all chemicals, including ethanol, were purchased from Beijing Chemical Works (Beijing, PR China).

### Ethanol exposure and probiotic administration

**Fig. 1:**
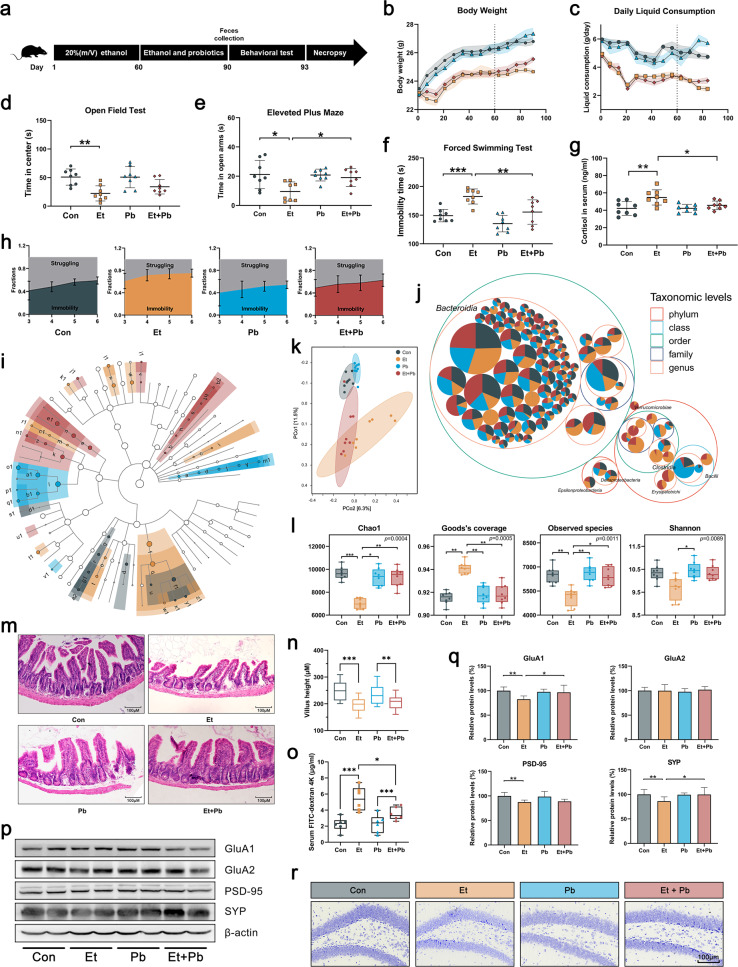
Probiotics alleviated CEE-induced depressive-like behavior and gut microbiota dysbiosis. **a** Experimental design. **b** Body weight of mice. **c** Daily liquid consumption of mice. **d** Time spent in the central area of the OFT. **e** Time spent in the open arms of the EPM. **f** Immobility time in the FST. **g** Serum cortisol concentration. **h** Dynamic motion state in the FST. **i** Linear discriminant analysis effect size (LEfSe) analysis showed the significantly enriched microbiome in each group (the legend is shown in Fig. S2b). **j** Taxonomic differences are based on 16S rRNA gene sequences extracted from the metagenome. **k** PCoA plot showing unweighted UniFrac distances, demonstrating significant changes in the gut microbiota after CEE and probiotic administration. **l** Probiotics attenuated CEE-induced changes in α-diversity. **m**, **n** Villus length with representative photomicrographs of ileal tissue (scale bar = 100 μm). **o** Intestinal permeability was detected by FD4 gavage. **p**, **q** Representative Western blot of synaptic proteins in the hippocampus and quantification. **r** Representative Nissl staining of the hippocampal dentate gyrus (DG) area (scale bar = 100 μm). Data are expressed as the mean ± SD, *n* = 8. *p < 0.05, **p < 0.01, ***p < 0.001.

The ethanol-free drinking model was established as described in previous reports [4, 22]. Mice were randomly divided into a control (Con) group, a 20% (m/V) ethanol (Et) group, a probiotics (Pb) group, and a probiotics +20% (m/V) ethanol (Et + Pb) group (Fig. 1a). Mice in the Et and Et + Pb groups received 10% ethanol for 2 days and 15% ethanol for 5 days, and then on day 8, their ethanol exposure was increased to the maximum concentration, which lasted for 90 days. Ethanol solutions or drinking water were updated once a day. From day 61, the animals were treated with either saline or BIOTICS (1 × 10^9^ cfu/ml; main components: *Lactobacillus rhamnosus*, *Lactobacillus acidophilus* and *Bifidobacterium*) at a dose of 200 μl once daily for the last 30 consecutive days.

### Microinjection of AAV and CEE

Four-week-old C57BL/6N mice were microinjected with con-shRNA-EGFP or NLRP3 shRNA-EGFP (4 μl of 10^12^ vg/ml, 200 nl/min, Interference sequence: CCG GCC GGC CTT ACT TCA ATC TGT TCT CGA GAA CAG ATT GAA GTA AGG CCG GTT TTT, HANBIO, Shanghai, China), which targeted the hippocampus, into the lateral ventricle using the following microinjection coordinates: 0.58 mm caudal of bregma, 1 mm right from the sagittal midline, and 1.85 mm deep from the skull surface [23]. Four weeks after microinjection, mice were divided into groups with or without 20% (m/V) ethanol exposure for 90 days. Mice in the Et group received 10% ethanol for 2 days and 15% ethanol for 5 days, and then on day 8, their ethanol exposure was increased to the maximum concentration, which lasted for 90 days.

### Fecal microbiota transplantation (FMT)

FMT was performed as described by Leclercq et al. [15] with some modifications. Recipient mice were orally gavaged with a broad-spectrum antibiotic cocktail and cleaning solution to clean the native microbiota. Each fecal sample from donor mice was suspended in sterile phosphate-buffered saline (PBS), and the suspension was immediately administered to the mice by oral gavage. Mice were fed for 2 weeks to stabilize the gut microbiota. More details are included in the Supplementary information: Supplementary Methods.

### Behavioral tests

Behavioral tests were conducted by two skilled operators who did not know which group the animals were in. Mice were acclimated to the testing room for 2 h before testing. Behavioral test data were recorded by the SMART™ tracking software program. Specific details are included in the Supplementary information: Supplementary Methods.

### Animal tissue extraction

The mice were anesthetized with isoflurane and then decapitated after cervical dislocation as previously reported [24]. The right hippocampus was separated and stored in a −80 °C freezer, and the left hippocampus was fixed for morphological examination.

### Determination of serum inflammatory cytokines

A selected profile of cytokines (IFN-γ, IL-1β, IL-2, IL-4, IL-5, IL-6, IL-10, IL-12p70, KC and TNF-α) was quantified in serum (diluted 1:4 in assay diluent) using Luminex Multiplex Assays according to the manufacturer’s instructions.

### 16S rRNA gene sequencing

Total bacterial DNA from fecal samples was isolated using a QIAamp DNA Stool Kit (Qiagen, Duesseldorf, Germany). The yield and quality of DNA were measured by a Nanodrop ND 1000 Spectrophotometer (Thermo Fisher Scientific) and 0.8% agarose gel electrophoresis, respectively. The V3–V4 region of the bacterial 16S rRNA gene was amplified by polymerase chain reaction (PCR) (forward primer: 5′-ACT CCT ACG GGA GGC AGC A-3′ and reverse primer: 5′-GGA CTA CHV GGG TWT CTA AT-3′). PCR products were purified with Vazyme VAHTS™ DNA Clean Beads (Vazyme, Shanghai, PR China) and quantified using a PicoGreen dsDNA Assay Kit (Invitrogen, CA, USA). The sequencing service was provided by Personal Biotechnology Co., Ltd. (Shanghai, PR China). Alpha diversity, including the Chao1 and Shannon indices, was calculated using operational taxonomic units (OTUs) in QIIME. Beta diversity was visualized by PCoA.

### WB and other experiments

WB, SDV, ELISA, real-time quantitative PCR, immunofluorescence, and Nissl staining were performed as described in the Supplementary information: Supplementary Methods.

### Intestinal permeability assay

Intestinal permeability was detected as previously described with some modifications [25]. After the last behavioral experiment, the mice were fasted for 4 h. The mice were gavaged with FITC-dextran mol wt 4000 (FD4) at 600 mg/kg body weight. Four hours later, the mice were sacrificed, and blood was collected to obtain serum. In total, 50 μl serum samples were diluted with 1× PBS. FITC-dextran concentrations were determined by an Infinite F200 PRO apparatus (TECAN, Switzerland) using wavelength settings of excitation at 485 nm and emission at 590 nm against a calibration curve of known concentrations.

### Causal mediation analysis

Causal mediation analysis was used to explore the mediation effect of inflammatory cytokines on the association between the gut microbiota and depressive-like behavior [26, 27]. We applied the *Z* score to quantify the performance of mice that received FMTs from NLRP3-shRNA groups in behavioral tests. The statistics included the time in the central area of the OFT (*Z*_(OFT)_), the time in the open arms of the EPM (*Z*_(EPM)_), the immobility time in the FST (*Z*_(FST)_), the immobility time in the tail suspension test (*Z*_(TST)_), and sucrose preference in the sucrose preference test (*Z*_(SPT)_):

1 $$Z_{(i)} = \frac{{x_i - \mu }}{S}$$

*μ*: mean; *S*: standard deviation.

Calculation of the overall *Z* score in behavioral tests:

2 $$Z = - Z_{({{{{{\rm{OFT}}}}}})} - Z_{({{{{{\rm{EPM}}}}}})} + Z_{({{{{{\rm{FST}}}}}})} + Z_{({{{{{\rm{TST}}}}}})} - Z_{({{{{{\rm{SPT}}}}}})}$$

Causal mediation analyses were carried out using R 4.1.0 with an R package (“mediation”) based on two general linear models (Supplementary Fig. 16): the mediator model and the outcome model. The total effect included the direct effect estimated and the indirect effect. The ratio of the indirect effect to the total effect was defined as the proportion of mediation, and 95% confidence intervals for mediated proportions were derived by the quasi-Bayesian Monte Carlo simulation for 1000 times. Detailed statistical data are provided in the Supplementary information: Raw data of causal mediation analysis.

### Statistical analysis

The minimal sample size was pre-determined by the nature of experiments. No statistical methods were used to pre-determine sample sizes, but our sample sizes are similar to those reported in previous publications [4, 27]. Details of the number of independent experiments are provided in the figure legends. For all experiments in this study, the animals were randomly assigned to experimental groups and control groups. Investigators involved in conducting experiments, collecting data and performing analyses were blinded to the mice groups.

Datasets were checked for normality, variations, and statistical tests using GraphPad Prism 8. All data are expressed as the mean ± standard deviation unless otherwise stated. Statistical analysis of data was performed using analysis of variance and Tukey′s multiple comparisons test. A *p* value of <0.05 was considered significant. The Genescloud tools (http://www.genescloud.cn), a free online platform for data analysis, and GraphPad Prism 8 were used for statistical analysis. Detailed statistical data are provided in the Supplementary information: Table S4.

## Results

### Probiotics alleviated CEE-induced depressive-like behavior, gut microbiota dysbiosis and neuron damage in the hippocampus

To explore the effects of ethanol on behavior and the gut microbiota, we established a CEE mouse model and added probiotics for comparison. After 90 days of ethanol exposure, mice in the ethanol group (Et group) showed a slower growth rate (Fig. 1b), lower fluid consumption (Fig. 1c), less time spent in the central area of the open field test (OFT) (Fig. 1d) and the open arms of the elevated plus maze (EPM) (Fig. 1e), increased immobility time and an early onset of immobility in the forced swim test (FST) (Fig. 1f, h), and higher serum cortisol levels (Fig. 1g) than mice in the control group (Con group), indicating that mice developed anxiety-like and depressive-like behavior and high stress levels. Compared with the Con group, mice in the probiotic group (Pb group) showed no significant behavioral or emotional effects. However, probiotics effectively attenuated anxiety-like behavior, depressive-like behavior and high stress levels in CEE-exposed mice.

16S rRNA gene sequencing revealed the composition of the gut microbiota in each group. The results showed significant differences in the composition and biomarkers of the gut microbiota among groups (Fig. 1i, j and Supplementary Fig. 2a, b). The heatmap showed that probiotic administration increased the abundance of *Lactobacillus* (Supplementary Fig. 2d). Principal coordinate analysis (PCoA) related to β-diversity showed a separation of normal-diet mice and CEE-exposed mice (Fig. 1k). The diversity and richness of the gut microbiota in the Et group were significantly lower than those in the other groups based on α-diversity (Fig. 1l and Supplementary Fig. 2c). These results suggest that CEE induced intense gut microbiota dysbiosis, which could be relieved by probiotics to some extent. In addition, probiotics had no significant effect on the total distance traveled in the OFT or blood ethanol concentration (Supplementary Fig. 1a, b), indicating that the above behavior and emotional changes were not caused by a loss of motor ability or blocked ethanol absorption.

Gut microbiota dysbiosis is accompanied by disruptions of intestinal homeostasis and the immune response. In line with these observations, we found a loss of intestinal homeostasis (reduced expression of Reg3g and Lcn2, Supplementary Fig. 3), intestinal structure atrophy (reduced villus height, Fig. 1m, n), increased intestinal permeability (Fig. 1o) and increased levels of serum lipopolysaccharide (LPS) and IL-1β in CEE-exposed mice. Probiotics led to no significant improvement in the disruption of intestinal structure, but they led to weak recovery of intestinal barrier function and reduced the increased levels of serum LPS and IL-1β.

We also detected hippocampal alterations, which are critical in the pathogenesis of depression, by Western blotting (WB) and Nissl staining. Synaptic proteins in the Et group showed lower expression (Fig. 1p, q), and Nissl bodies in the Et group were reduced, had unclear stratification, and manifested an irregular and loose arrangement (Fig. 1r and Supplementary Fig. 3c), indicating neuron damage in the hippocampus induced by CEE. Probiotics effectively protected hippocampal neurons from CEE-induced damage.

### FMT from CEE-exposed mice induced depressive-like behavior and hippocampal neuroinflammation in recipient mice

**Fig. 2:**
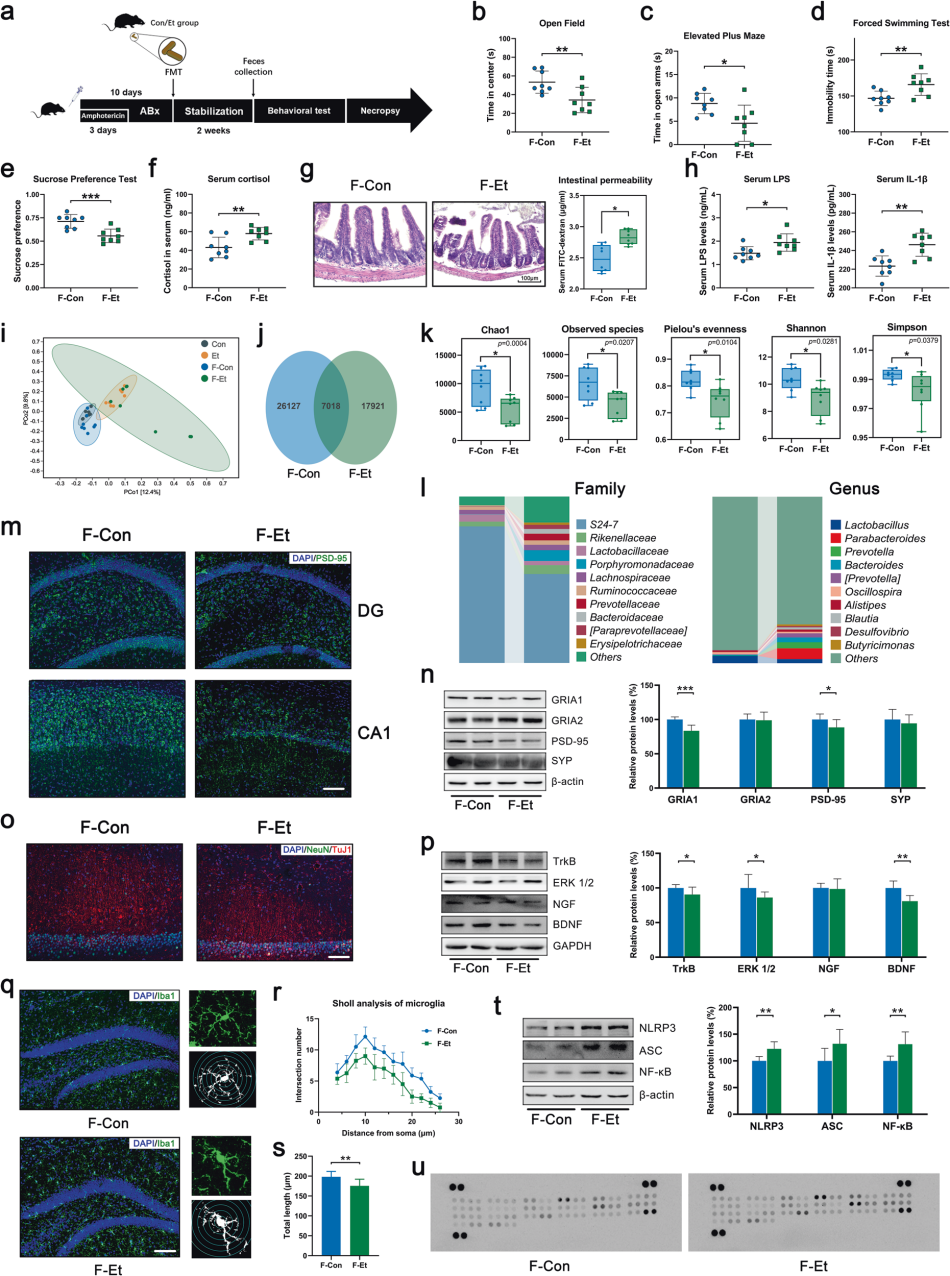
FMT from CEE-exposed mice induced depressive-like behavior and hippocampal neuroinflammation in recipient mice. **a** Experimental design of FMT. **b** Time in the central area of the OFT. **c** Time spent in the open arms of the EPM. **d** Immobility time in the FST. **e** Immobility time in the TST. **f** Cortisol concentration in mouse serum. **g** Representative photomicrographs of ileum tissue and intestinal permeability (scale bar = 100 μm). **h** Serum LPS and IL-1β concentrations. **i** PCoA plot showing unweighted UniFrac distances between donor mice and recipient mice. **j** Venn diagram of the OTUs detected in the recipient mice. **k** Difference in α-diversity among recipient mice. **l** Barplots showing the relative abundance of the gut microbiota at the family and genus levels. **m**, **n** IF and Western blot showing that FMT from CEE-exposed mice reduced the expression of synaptic proteins in the hippocampus (scale bar = 100 μm). **o** IF of NeuN and TuJ1 showing the state of neurons in the hippocampal CA1 area (scale bar = 100 μm). **p** Western blot showing that FMT from CEE-exposed mice reduced the expression of neurotrophic proteins in the hippocampus. **q**–**s** IF of Iba1 and Sholl analysis showing the number, morphology, and length of microglia in the hippocampus (scale bar = 100 μm). **t** FMT from CEE-exposed mice reduced the expression of NLRP3, ASC and NF-κB in the hippocampus. **u** Mouse cytokine array showed an increase in 18 inflammatory factors or chemokines in the hippocampus after FMT from CEE-exposed mice (the legend is shown in Fig. S7). Data are expressed as the mean ± SD, *n* = 8. *p < 0.05, **p < 0.01, ***p < 0.001.

To exclude the direct effects of ethanol on the brain and intestinal tract, we transplanted the gut microbiota of the mice in the Con and Et groups into healthy recipient mice (Fig. 2a). We first cleaned the native microbiota of recipient mice with an antibiotic cocktail (Supplementary Fig. 4a–d) and quantified the ethanol content of the transplant filtrate (Supplementary Fig. 4e). PCoA showed that the gut microbiota of recipient mice clustered with that of their donor (Fig. 2i). The results strongly support that the direct effect of ethanol had been excluded, while the effect of the microbiota had been transferred.

Mice that received CEE-exposed donor gut microbiota showed less time spent in the central area of the OFT and open arms of the EPM, increased immobility time in the FST, a reduced sucrose preference index and increased serum cortisol compared with mice that received control donor gut microbiota (Fig. 2b–f and Supplementary Fig. 5), indicating that mice developed anxiety-like and depressive-like behavior and high stress levels. After the subdiaphragmatic vagotomy (SDV) protocol, the anxiety-like behavior in recipient mice induced by FMT was alleviated, but it had no significant effect on the immobility time in FST (Supplementary Fig. 15).

16S rRNA gene sequencing showed significant differences in the composition and biomarkers of the gut microbiota among groups (Fig. 2i–l and Supplementary Fig. 6b, c). FMT from CEE-exposed mice obviously reduced the diversity and richness of the gut microbiota, which was similar to that in donor mice (Fig. 2k and Supplementary Fig. 6a).

FMT from CEE-exposed mice also increased intestinal barrier permeability (Fig. 2g) and serum inflammatory factor levels (Fig. 2h), and reduced the expression of synaptic proteins and neurotrophic proteins in the hippocampus (Fig. 2m, n, p). NeuN and TuJ1 staining showed a loss of neurons and shorter neuronal axons in the CA1 area of the hippocampus (Fig. 2o). These results showed that hippocampal neuronal injury was also transferred to the recipient mice along with the gut microbiota.

We further detected neuroinflammation in the hippocampal area to explore the possible causes of neuronal damage. FMT from CEE-exposed mice induced the activation of microglia (the number and cell body size of microglia increased, whereas cell complexity and total length of microglial processes decreased, Fig. 2q–s) and the NLRP3 inflammasome (increased expression of NF-κB, NLRP3 and ASC, Fig. 2t) and a significant immune response (18 of 40 inflammatory cytokines or chemokines, Fig. 2u and Supplementary Fig. 7) in the hippocampus. The results of FMT strongly suggest that the gut microbiota regulates CEE-induced depressive-like behavior and that NLRP3-mediated neuroinflammation may play an important role in this process.

### Downregulation of hippocampal NLRP3 expression alleviated CEE-induced depressive-like behavior and neuronal injury

**Fig. 3:**
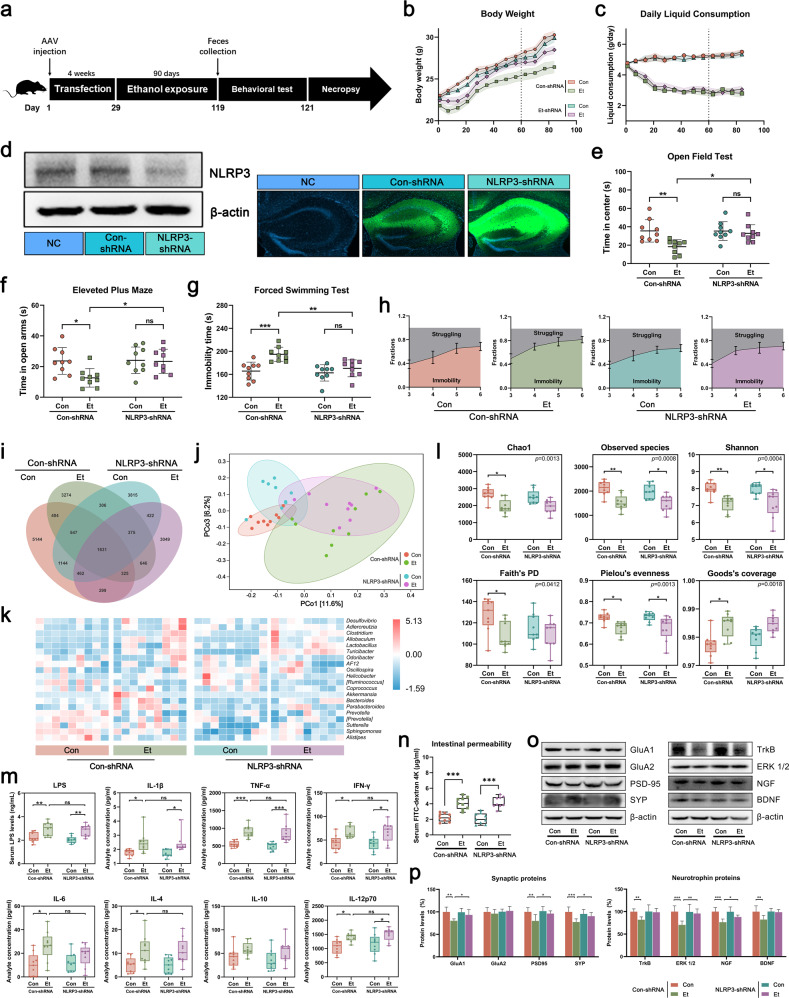
Effects of hippocampal NLRP3 in CEE-exposed mice. **a** Experimental design for AAV transfection and CEE administration. **b** Body weight of mice. **c** Daily liquid consumption of mice. **d** Effect of GFP-labeled AAV transfection on reducing hippocampal NLRP3 expression. **e** Time spent in the central area of the OFT. **f** Time spent in the open arms of the EPM. **g** Immobility time in the FST. **h** Dynamic motion state in the FST. **i** Venn diagram of the OTUs. **j** PCoA plot showing unweighted UniFrac distances among transfected mice. **k** Heatmap showing the distribution trends of species abundance in each sample. **l** Difference in α-diversity among transfected mice. **m** Serum LPS and inflammatory cytokine concentrations detected by ELISA and Luminex assays. **n** Intestinal permeability was detected by FD4 gavage. **o**, **p** Expression of synaptic proteins and neurotrophic proteins in the hippocampus of transfected mice. Data are expressed as the mean ± SD, *n* = 9. *p < 0.05, **p < 0.01, ***p < 0.001.

To clarify the specific mechanism by which the gut microbiota regulates CEE-induced depressive-like behavior, we downregulated hippocampal NLRP3 expression with AAV transfection (Fig. 3a, d). AAV could limit its effects on the hippocampus and prevent the peripheral NLRP3-mediated immune response from affecting the gut microbiota. CEE induced slower growth (Fig. 3b), anxiety-like and depressive-like behavior (less time spent in the central area of the OFT and open arms of the EPM, increased immobility time and an early onset of immobility in the FST, Fig. 3e–h), and neuronal damage in the hippocampus (reduced expression of synaptic proteins and neurotrophic proteins, Fig. 3o, p) only in the mice transfected with Con-shRNA but not NLRP3-shRNA. These results indicated that hippocampal NLRP3 is critical to CEE-induced depressive-like behavior. 16S rRNA gene sequencing showed that the downregulation of hippocampal NLRP3 had no significant effect on liquid consumption (Fig. 3c), the intestinal barrier (Fig. 3n and Supplementary Fig. 11a) and diversity of the gut microbiota (Fig. 3j, l and Supplementary Fig. 10a) but changed the abundance of some OTUs (Fig. 3i, k and Supplementary Fig. 10b–d).

Considering that peripheral inflammation is important for the activation of the NLRP3 inflammasome, we performed a more comprehensive detection of serum LPS and inflammatory cytokines by ELISA and Luminex assays. The results showed that CEE induced the elevation of LPS and various inflammatory cytokines (IL-1β, TNF-α, IFN-γ, IL-6, IL-4, and IL-12p70, Fig. 3m) in serum, but the downregulation of hippocampal NLRP3 expression had no significant effect on these cytokines. These results suggest that circulating inflammatory factors are of peripheral origin and not the result of hippocampal NLRP3 inflammasome activation.

### FMT from hippocampal NLRP3-downregulated mice induced depressive-like behavior and systemic inflammation in recipient mice

**Fig. 4:**
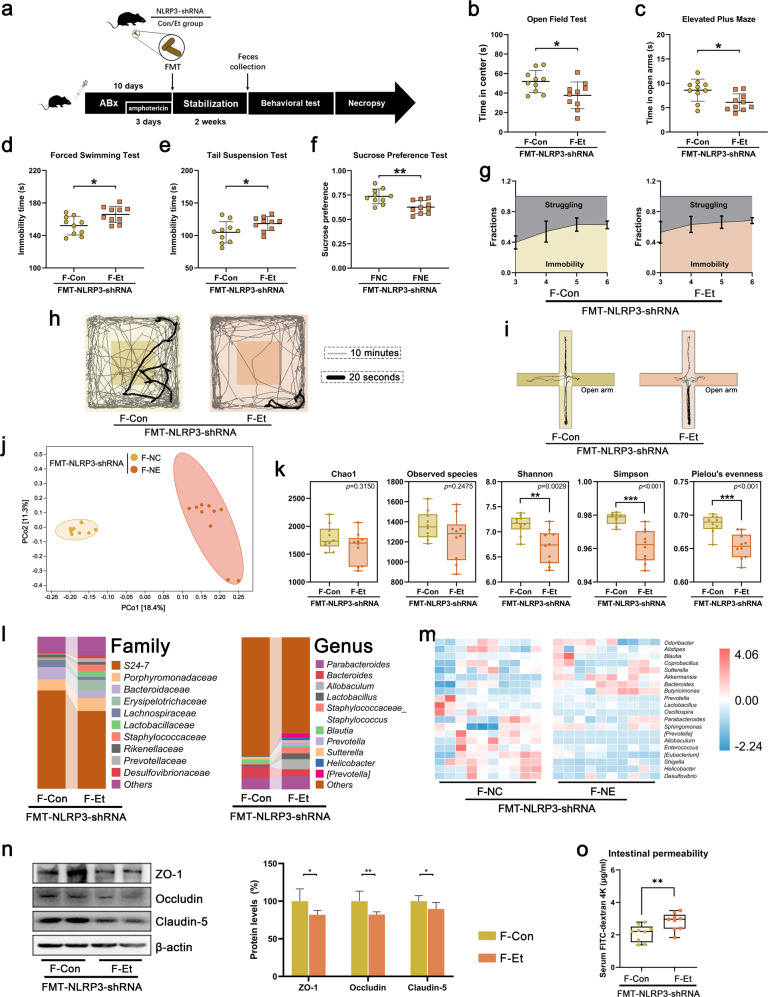
FMT from hippocampal NLRP3-downregulated mice induced depressive-like behavior in recipient mice. **a** Experimental design for FMT from hippocampal NLRP3-downregulated mice. **b** Time in the central area of the OFT. **c** Time spent in the open arms of the EPM. **d** Immobility time in the FST. **e** Immobility time in the TST. **f** Sucrose preference in the SPT. **g** The dynamic motion state in the FST. **h** Representative tracks of recipient mice in the OFT. **i** Representative tracks of recipient mice in the EPM. **j** PCoA plot showing unweighted UniFrac distances among recipient mice. **k** Difference in α-diversity among recipient mice. **l** Barplots showing the relative abundance of the gut microbiota at the family and genus levels. **m** Heatmap showing the distribution trends of species abundance in each sample. **n** Western blot showing the expression of tight junction proteins in ileal tissue. **o** Intestinal permeability was detected by FD4 gavage. Data are expressed as the mean ± SD, *n* = 10. *p < 0.05, **p < 0.01, ***p < 0.001.

**Fig. 5:**
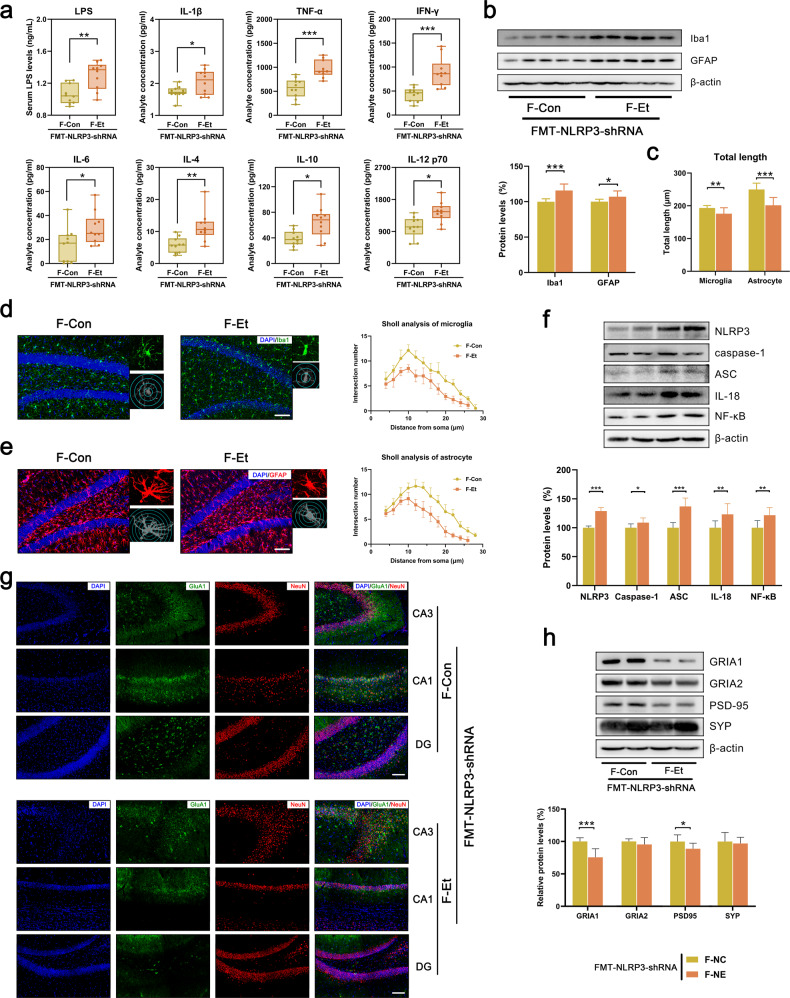
FMT from hippocampal NLRP3-downregulated mice induced systemic inflammation in recipient mice. **a** Serum LPS and inflammatory cytokine concentrations in recipient mice detected by ELISA and Luminex assays. **b** Western blot showing the expression of Iba1 and GFAP in the hippocampus of recipient mice. **c**–**e** IF of Iba1/GFAP and Sholl analysis showing the number, morphology, and length of microglia/astrocytes in the hippocampus (scale bar = 100 μm). **f** Western blot showing the expression of NLRP3, caspase-1, ASC, IL-18 and NF-κB. **g**, **h** Western blot and IF showing the expression of synaptic proteins in the hippocampus (scale bar = 100 μm). Data are expressed as the mean ± SD, *n* = 10. *p < 0.05, **p < 0.01, ***p < 0.001.

The gut microbiota from two groups of hippocampal NLRP3 downregulated mice were subsequently transplanted into healthy recipient mice for further study (Fig. 4a). Consistent with the donor mice, there were significant differences in the composition of the gut microbiota (reduced diversity and richness and different abundance of OTUs, Fig. 4j–m and Supplementary Fig. 12d–g), the structure and function of the intestinal barrier (intestinal permeability and the expression of tight junction proteins in ileum tissue, Fig. 4n, o), as well as serum LPS and inflammatory cytokines (Fig. 5a) between the two groups of recipient mice. Although hippocampal NLRP3-downregulated mice did not exhibit anxiety or depression after ethanol exposure, their gut microbiota still had the ability to cause anxiety-like and depressive-like behavior (Fig. 4b–i and Supplementary Fig. 12a–c), the activation of the NLRP3 inflammasome (increased expression of NLRP3, Caspase-1, ASC, IL-18 and NF-κB, Fig. 5f), neuroinflammation (the activation of microglia and astrocytes, Fig. 5b–e) and neuronal injury (reduced number of neurons and reduced expression of synaptic proteins and neurotrophic proteins, Fig. 5f–h and Supplementary Fig. 14) in the hippocampus. These results further confirmed that hippocampal NLRP3 is critical for the gut microbiota to regulate CEE-induced depressive behavior and that the gut microbiota is a source of peripheral inflammatory cytokines.

### LPS and inflammatory cytokines mediate the association between the gut microbiota and depressive-like behavior

**Fig. 6:**
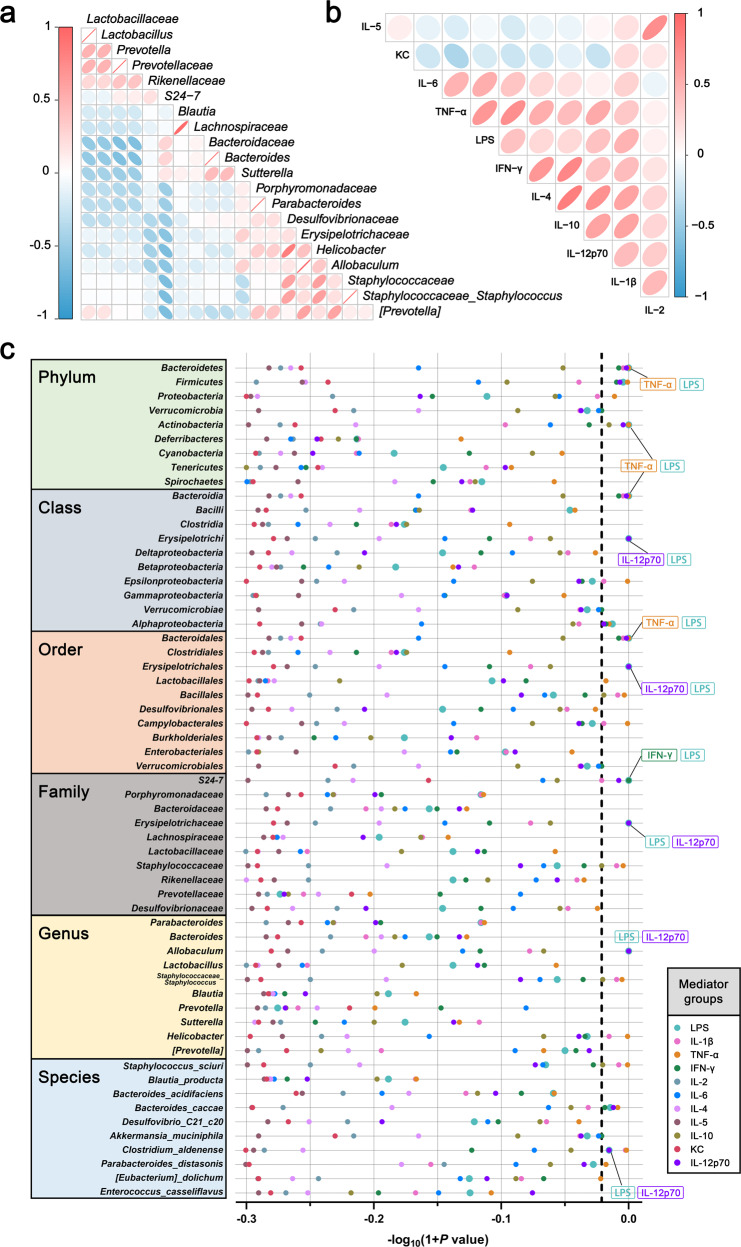
LPS and inflammatory cytokines mediate the association between the gut microbiota and depressive-like behavior. **a** Correlation between the gut microbiota. **b** Correlation between LPS and inflammatory cytokines. **c** Estimated −log_10_(*p* values) of natural indirect effects from pairwise weighted mediation models (the mediation model is shown in Fig. S14) for depressive-like behavior. The dashed vertical line signifies a *p* value threshold of 0.05, and the points on the right side of the line estimates are below that threshold. Overlapping points have been labeled.

Inflammatory cytokines are an important factor in hippocampal NLRP3 inflammasome activation. To confirm whether the gut microbiota activates the hippocampal NLRP3 inflammasome through peripheral inflammatory factors and to explore which species are involved, causal mediation analysis was introduced into this study based on the recipient mice that received FMTs from NLRP3-shRNA groups. Mice with elevated abundance of *Firmicutes*, *Actinobacteria*, *Erysipelotrichi*, *Erysipelotrichales*, *Bacillales*, *Erysipelotrichaceae*, *Staphylococcaceae*, *Allobaculum*, *Staphylococcaceae_ Staphylococcus*, *Helicobacter*, and *Clostridium_ aldenense* and reduced abundance of *Bacteroidetes*, *Bacteroidia*, *Verrucomicrobiae*, *Bacteroidales*, *S24-7*, and *Bacteroides_caccae* had an increased risk of depressive-like behavior, and LPS, IL-1β, TNF-α, IFN-γ and IL-12p70 mediated this effect (Fig. 6a–c).

## Discussion

Gut microbiota alterations have been reported to be associated with ethanol-induced psychological symptoms, including depression, alcohol craving and social rejection.

In this study, we first demonstrated that probiotics protect mice from CEE-induced depressive-like behavior and hippocampal neuronal injury. Probiotic administration selected *Lactobacillus rhamnosus*, *Lactobacillus acidophilus* and *Bifidobacterium*, which are reported to be effective in alleviating depressive syndromes caused by chronic stress [28, 29]. Hippocampal changes play an important role in the pathogenesis of depression. For example, reduced hippocampal volume is a marker of clinical depression [30]. Our previous studies have also shown that the depression-like behavior induced by CEE is associated with damage to hippocampal neurons [8]. The protective effect of probiotics fully suggests that the gut microbiota may be involved in CEE-induced depressive disorder.

Ethanol can cross the blood–brain barrier freely and be evenly distributed in multiple organs within minutes of drinking alcohol [31]. The FMT method was used to remove the direct effects of ethanol on the brain. Consistent with our hypothesis, CEE-induced depression-like behavior and hippocampal neuronal damage were both transferred to healthy recipient mice. Overall, our FMT experiment revealed that CEE-induced depressive-like behavior is regulated by the gut microbiota. We tried to elucidate the mechanisms linking the microbiota to hippocampal injury and depressive behavior.

A number of studies have established that the vagus nerve plays an important role in gut-brain connection in depressive models [32–35]. However, in the present study, after transection of the vagus nerve in recipient mice, there was no significant improvement in FMT-induced depressive-like behavior, and only a slight relief in anxiety-like behavior was observed. This suggests that there is a more important mechanism, besides the vagus nerve, by which gut microbiota causes depressive-like behavior in mice after CEE.

Interestingly, we found that FMT from CEE-exposed mice led to serious hippocampal neuroinflammation and the activation of the NLRP3 inflammasome. NLRP3 is activated by a variety of pathogen-associated molecular patterns or damage-associated molecular patterns and mediates host immune responses to microbial infection or cellular damage [36]. The NLRP3 inflammasome has been implicated in the development of several neurological disorders, including Alzheimer’s disease and depression [37]. Multiple antidepressants have been shown to inhibit NLRP3 inflammasome activation [38, 39]. Mice with NLRP3 gene knockout (KO) did not exhibit depressive-like behavior after chronic unpredictable mild stress (CUMS) stimulation [40]. Lowe et al. showed that treatment with an antibiotic cocktail reduced ethanol-induced activation of the NLRP3 inflammasome and neuroinflammation in the hippocampus and cortex [21]. Based on these findings, we hypothesized that hippocampal NLRP3 inflammasome-mediated neuroinflammation is responsible for gut microbiota-induced neuronal damage and depressive behavior after CEE.

To verify the effect of NLRP3, AAV microinjection was used to downregulate the expression of NLR3 in the hippocampus. Compared with NLRP3 KO mice, AAV microinjection can effectively prevent the peripheral immune response and gut microbiota of mice from being affected. NLRP3 or Caspase-1 KO led to an imbalance in the Firmicutes/Bacteroidetes ratio in mice and interfered with the intestinal immune response [41, 42]. It has been reported that the gut microbiota from NLRP3 KO mice could ameliorate CUMS-induced depressive-like behavior [43]. AAV-transfected mice did not exhibit depressive-like behavior or hippocampal neuronal damage after ethanol exposure, confirming that NLRP3 is critical for ethanol-induced depressive disorder.

The priming signals for NLRP3 activation are provided by microbial components or endogenous cytokines, leading to the activation of the transcription factor NF-κB and subsequent upregulation of NLRP3 and pro-IL-1β. Activation signals are provided by a variety of stimuli, including extracellular ATP, pore-forming toxins, and particulate matter [36]. In this study, mice that received CEE or FMTs showed increased LPS and IL-1β levels in serum, which were positively correlated with the immobility time in the FST. Currently, the gut microbiota is thought to be a major source of inflammation in patients with MDD. The interaction between the gut microbiota and intestinal mucosa regulates the production of proinflammatory cytokines or chemokines, including IL-8, IL-10 and TGF-β [44]. The alteration of the gut microbiota could also activate the peripheral immune system by stimulating immune cells, including dendritic cells and T cells, to release proinflammatory cytokines [45, 46]. In addition, reports have suggested that gut microbiota metabolites disrupt the intestinal barrier and increase its permeability, allowing bacterial components and inflammatory cytokines to enter the circulation [47]. Therefore, the Luminex method was used for a more comprehensive quantitative detection of inflammatory cytokines in the serum of mice. CEE caused an obvious increase in serum inflammatory factors, but hippocampal NLRP3 downregulation had no significant effects on these changes. The results showed that the elevated serum inflammatory factors in CEE mice were of peripheral origin rather than the result of the activation of hippocampal NLRP3.

Although hippocampal NLRP3-downregulated mice did not show depressive-like behavior or hippocampal damage after ethanol exposure, CEE-induced peripheral changes still existed, and the fecal microbiota could still induce depressive-like behavior in recipient mice. The recipient mice were highly similar to those that received FMTs from untransfected mice in terms of gut microbiome composition, the intestinal barrier, serum inflammatory cytokines, hippocampal neuroinflammation and neuronal damage. These results suggest that gut microbiota alteration is involved in CEE-induced elevation of peripheral inflammatory cytokines. Elevated peripheral cytokines can be actively transported to the central nervous system and lead to the activation of microglia and astrocytes [48, 49]. In a meta-analysis, microglial activity was associated with increased levels of IL-6, IL-8, and TNF-α in the brain parenchyma or cerebrospinal fluid of patients with MDD [50]. Since it is difficult to target a single microbiome species or inflammatory factors with existing methods, we introduced causal mediation analysis, which is widely used in epidemiological studies, to analyze the associations among the gut microbiota, inflammatory cytokines and depressive-like behavior. The results confirmed the mediation effect of LPS, IL-1β, TNF-α, IFN-γ and IL-12.

In summary, CEE induced gut microbiota dysbiosis and intestinal homeostasis disruption. The alteration of the gut microbiota led to increased LPS and inflammatory cytokines in the circulation and then activated the NLRP3 inflammasome in the hippocampus, leading to neuroinflammation and depressive-like behavior (Supplementary Fig. 17). Our results reveal a new mechanism of CEE-induced depressive disorder and provide a new therapeutic direction for the prevention and treatment of ethanol-related psychiatric disorders.

## Supplementary information

Supplemental information

Raw data of causal mediation analysis

## Data Availability

The raw data of 16S rRNA sequencing were deposited in the NCBI Sequence Read Archive (SRA) database under accession number PRJNA826079, PRJNA826215, PRJNA826233.
