# Supplementary material for: Gut microbiota regulates chronic ethanol exposure-induced depressive-like behavior through hippocampal NLRP3-mediated neuroinflammation
Source: Mol Psychiatry. 2022 Oct 24;28(2):919–30. doi: 10.1038/s41380-022-01841-y (PMC9908543; doi:10.1038/s41380-022-01841-y)

Supplemental information for

**Gut microbiota regulates chronic ethanol exposure-induced depressive-like behavior through hippocampal NLRP3-mediated neuroinflammation**

Hui Yao^#^, Dalin Zhang^#^, Hao Yu, Huiya Yuan, Hui Shen, Xinze Lan, Hao Liu, Xiaohuan Chen, Fanyue Meng, Xu Wu, Guohua Zhang^*^, Xiaolong Wang^*^

^#^These authors contributed equally to the work. ^*^Corresponding authors.

wangxiaolong@cmu.edu.cn (X. Wang); ghzhang@cmu.edu.cn (G. Zhang).

**The PDF file includes:**

**Supplementary methods**

**Supplementary Table. 1** Key resources table

**Supplementary Table. 2** Primer sequences used for verification of ABx administration before FMT

**Supplementary Table. 3** Primer sequences used for RT-qRCR

**Supplementary Table. 4** Statistical data

**Supplementary Fig. 1** Blood ethanol concentration, supplementary data of behavioral tests, and representative cortisol LC-MS chromatograms. *Related to* ***Fig. 1***

**Supplementary Fig. 2** Supplementary data of 16S rRNA gene sequencing. *Related to* ***Fig. 1***

**Supplementary Fig. 3** Supplementary data about ileum, serum and hippocampus. *Related to* ***Fig. 1***

**Supplementary Fig. 4** Preparation for FMT: cleaning of gut microbiota and examination of filtrate for transplantation. *Related to* ***Fig. 2***

**Supplementary Fig. 5** Supplementary data of behavioral tests, and representative cortisol LC-MS chromatograms. *Related to* ***Fig. 2***

**Supplementary Fig. 6** Supplementary data of 16S rRNA gene sequencing. *Related to* ***Fig. 2***

**Supplementary Fig. 7** The legend of mouse cytokine array. *Related to* ***Fig. 2u***

**Supplementary Fig. 8** AAV transfection had no effect on expression of NLRP3 in ileum tissue. *Related to* ***Fig. 3d***

**Supplementary Fig. 9** Supplementary data of behavioral tests. *Related to* ***Fig. 3***

**Supplementary Fig. 10** Supplementary data of 16S rRNA gene sequencing. *Related to* ***Fig. 3***

**Supplementary Fig. 11** Expression of tight junction proteins in ileum tissue, supplemental data of serum inflammatory cytokines. *Related to* ***Fig. 3***

**Supplementary Fig. 12** Supplementary data of behavior tests and 16S rRNA gene sequencing. *Related to* ***Fig. 4***

**Supplementary Fig. 13** Supplementary data of serum inflammatory cytokines detected by Luminex. *Related to* ***Fig. 5a***

**Supplementary Fig. 14** FMT from hippocampal NLRP3 down-regulated mice reduced expression of hippocampal neurotrophic proteins in recipient mice. *Related to* ***Fig. 5***

**Supplementary Fig. 15** Effects of subdiaphragmatic vagotomy (SDV) on behavior of recipient mice.

**Supplementary Fig. 16** The mediation model used in Fig. 6c. *Related to* ***Fig. 6c***

**Supplementary Fig. 17** Graphic abstract

**Supplementary Fig. 18** Raw images of western blots from all Figures

**Supplementary methods**

**Fecal microbiota transplantation (FMT)**

Recipient mice were gavaged with a broad-spectrum antibiotic cocktail (ampicillin, metronidazole, neomycin sulfate: 100 mg/kg; vancomycin: 50mg/kg) once a day for 10 days before FMT. Additional amphotericin-B (1 mg/kg) was added for the first 3 days to inhibit fungal growth. Then mice were subjected to intestinal purge with cleaning solution (PEG4000: 59 g/L; NaCl: 1.46 g/l; KCl: 0.75 g/l; Na2SO4: 5.68 g/l; NaHCO3: 1.68 g/l) in order to wash out the antibiotics remaining in the intestine. The cleaning solution was administered three times by oral gavage to each mouse (2× 500μL the day before FMT with 1 hour interval, and 1× 500 μL 4h before the FMT. Mice were fasted 4 h before cleaning solution administration. Since mice are coprophagous, the bedding was renewed after each intestinal purge. Each fecal sample was suspended in sterile PBS (1g sample with 10 ml PBS), then vortexed for 5 min followed by sedimentation for 5 min. The slurry was then passed through a 100 μM cell strainer and 200 μL of the suspension was immediately administered to the mice by oral gavage.

**Subdiaphragmatic vagotomy (SDV)**

As previously described^1^, SDV was performed under anesthesia with 5% isoflurane. A 1-cm right transverse abdominal incision was made 0.5 cm below the xiphisternum, starting from the linea alba. The liver was carefully retracted with a small cotton pellet dampened with sterile normal saline, and the vagus nerve was exposed and severed. For the sham grop, the trunk of the vagus nerve was gently exposed but not cut. The mice received SDV were allowed to recover for 2 weeks.

**Behavioral tests**

Open field test (OFT)

The OFT was used as previously reported to evaluate the depressive-like behaviors of mice^2^. The OFT consisted of an empty square arena (40×40×30 cm) constructed of plastic with a white base. The central region is 20×20 cm. Mice were placed individually in the corner of the OFT apparatus, and spontaneous activities were recorded for 10 min using the SMART™ tracking program. After each test, the arena was cleaned with 75% ethanol to eliminate odor cues.

Elevated plus-maze (EPM)

The EPM test was carried out as previously reported to evaluate the anxiety-like behaviors of mice^3^. Mice were tested in a cross-shaped maze consisting of two open arms (50×10 cm), two closed arms (50×10 cm), and a central region (10×10 cm). Each mouse was placed in the central region of the maze and allowed to explore for 5 min. The time spent in each arm was recorded using the SMART™ tracking program. After each test, the arena was cleaned with 75% ethanol to eliminate odor cues.

Forced swimming test (FST)

As previously reported^4^, the FST was performed to evaluate the depressive-like behavior. The test device consisted of a transparent cylindrical glass container (10 cm in diameter, depth of 22 cm) filled with water (23 °C to 25 °C) and a video camera in front of the container. The mice could not touch the bottom of the container with their hind legs. The test was conducted for 6 min: the first 2 min was an adaptation phase, after which the immobility of the mouse in the water was recorded for 4 min (Immobility refers to the mouse’s body floating with the absence of any movement except for those necessary for keeping the nose above water).

Tail suspension test (TST)

The TST test was performed as previously described to assess depressive-like behavior^5^. Mice were suspended by their tail (50 cm distance from the floor) using adhesive tape at 1 cm from the tip of the tail. The TST test was conducted for 6 min, and the duration of immobility in the last 4 min was recorded. TST data were recorded by the SMART™ tracking software program.

Sucrose preference test (SPT)

SPT was used to detect the anhedonia in mice. One day before the test, mice were given two identical water bottles, respectively containing pure water or 1% (w/v) sucrose solution. The bottles were switched every eight hours. The test lasted for 24 hours, and the weight of the bottle was measured at the beginning and end of SPT.

**Western blotting**

The extracted tissue was detergent-extracted on ice using radioimmunoprecipitation assay (RIPA) lysis buffer (Beyotime, Shanghai, P R China) with 1 mM phenylmethanesulfonyl fluoride (PMSF, Beyotime), disrupted on ice for 30 min, and then fragmented with ultrasonication. The lysates were collected and centrifuged at 21,000 *g* for 15 min. Total proteins were quantified using a BCA protein assay kit (Beyotime).

Equal amounts of protein (up to 30 μg) were separated by 10% SDS–PAGE and transferred to PVDF membranes. Transferred blots were blocked with nonfat milk for 2 hours and then incubated overnight at 4 °C with a primary antibody (1:2000). The primary antibodies are listed in Table. S1. Blots were subsequently washed and incubated with secondary antibodies for 2 hours. Protein bands were detected with an ECL reagent (Merck Millipore). Chemiluminescent signals were detected and analyzed using a Tanon-5500 chemiluminescent imaging system (Tanon Science and Technology Co., Ltd., Shanghai, P R, China). The intensity of the bands was analyzed using ImageJ 1.49 software (National Institutes of Health, Bethesda, MD, USA).

**Enzyme-linked immunosorbent assay (ELISA)**

The serum cortisol concentration of mice was determined using an ELISA kit (Table. S1) following the manufacturer’s instructions. Each sample (5× dilution) was used in 50 μl for detection, and the absorbance at 450 nm was measured. The concentration was calculated according to the standard curve drawn by Curve Expert 1.4 software.

**Nissl staining**

Mouse brain tissues were fixed with 4% paraformaldehyde overnight. After dehydration, the brain tissues were embedded in paraffin and then cut into 4-μm sections on a microtome (Leica) followed by Nissl staining. The sections were rinsed with Nissl Staining Solution (E607316, Sangon Biotech) for 20 min. The slides were dewaxed and stained in cresyl violet solution for 20 min, rinsed with ddH_2_O, decolorized in 75% ethanol, cleared in xylene, and sealed with neural resin. The sections were observed using a Zeiss Axio Scan Z1 automatic digital slide scanning system (Zeiss, Oberkochen, Germany).

**RT-qPCR**

Total mRNA was extracted from the mouse hippocampus using TRIzol (Thermo Fisher Scientific) and diluted to 200 ng/μl with RNase-free water. RNA was then reverse transcribed into cDNA using the PrimeScript™ RT Reagent kit. qPCR was performed using a SYBR® Premix Ex Taq II in a reaction volume of 20 μl with a Roche LightCycler 480 (Roche Diagnostics GmbH, Mannheim, Germany) using the following thermocycling conditions: 95 °C for 30 s, followed by 40 cycles of 95 °C for 5 s and 60 °C for 34 s, 95 °C for 15 s, 60 °C for 60 s and 95 °C for 15 s. Primer sequences are shown in Table. S3. The relative differences in expression between groups are expressed using cycle time (2^-ΔΔCt^) values and normalized to β-actin.

**Immunofluorescence**

30-μm thick brain tissue sections were washed with PBS. Then the sections were blocked by 8% BSA for 2 h and then treated overnight at 4 °C with primary antibody (1:200). Brain sections were subsequently washed and incubated with Alexa Flour 488/594 (1:400) for 2 h. Sections were washed and incubated with DAPI for 5 min. After washing, the sections were transferred to slides, and glass coverslips were mounted using mounting medium. Images were captured using Leica TCS SP8 laser scanning confocal microscope.

**References**

1. Pu, Y. et al. A role of the subdiaphragmatic vagus nerve in depression-like phenotypes in mice after fecal microbiota transplantation from Chrna7 knock-out mice with depression-like phenotypes. *Brain Behav. Immun.* **94**, 318-326 (2021). doi: 10.1016/j.bbi.2020.12.032.

2. Yao, H. et al. Chronic ethanol exposure induced depressive-like behavior in male C57BL/6 N mice by downregulating GluA1. *Physiol. Behav.* **234**, 113387 (2021). doi: 10.1016/j.physbeh.2021.113387.

3. Wang, X. et al. Chronic ethanol exposure induces neuroinflammation in H4 cells through TLR3 / NF-kappaB pathway and anxiety-like behavior in male C57BL/6 mice. *Toxicology*. **446**, 152625 (2020). doi: 10.1016/j.tox.2020.152625.

4. Yao, H. et al. AMPAkine CX516 alleviated chronic ethanol exposure-induced neurodegeneration and depressive-like behavior in mice. *Toxicol Appl Pharmacol*. **439**, 115924 (2022). doi: 10.1016/j.taap.2022.115924.

5. Yao, H. et al. Preventive effects of the AMPA receptor potentiator LY450108 in an LPS-induced depressive mouse model. *Behav. Brain Res.* **424**, 113813 (2022). doi: 10.1016/j.bbr.2022.113813.

**Supplemental tables**

**Supplementary Table. 1:** Key resources table.

| **REAGENT or RESOURCE** | **PRODUCERS** | **IDENTIFIER** |
| --- | --- | --- |
| **Antibodies** | | |
| Rabbit anti-ASC | Proteintech, Rosemont, IL, USA | 10500-1-AP; RRID:AB_2174862 |
| Rabbit anti-BDNF | Proteintech | 28205-1-AP; RRID:AB_2818984 |
| Rabbit anti-Claudin-5 | ZEN-BIO, Chengdu, P R China | 389414 |
| Rabbit anti-ERK1/2 | Proteintech | 16443-1-AP; RRID:AB_10603369 |
| Rabbit anti-GRIA1 | Merck Millipore, Billerica, MA, USA | AB1504; RRID:AB_2113602 |
| Rabbit anti-GRIA2 | Abcam, Cambridge, MA, USA | ab133477; RRID:AB_2620181 |
| Rabbit anti-Iba1 | Wako, Osaka, Japan | 019-19741; RRID:AB_839504 |
| Rabbit anti-NeuN | Abcam | ab177487; RRID:AB_2532109 |
| Rabbit anti-NF-κB | Proteintech | 10745-1-AP; RRID:AB_2178878 |
| Rabbit anti-NGF | Sangon Biotech, Shanghai, P R China | D260067 |
| Rabbit anti-NLRP3 | Proteintech | 19771-1-AP; RRID:AB_10646484 |
| Rabbit anti-Occludin | Abcam | ab168986; RRID:AB_2744671 |
| Rabbit anti-PSD-95 | Proteintech | 20665-1-AP; RRID:AB_2687961 |
| Rabbit anti-Synaptophysin | Proteintech | 17785-1-AP; RRID:AB_2271365 |
| Rabbit anti-TrkB | Proteintech | 13129-1-AP; RRID:AB_2155156 |
| Rabbit anti-ZO-1 | Abcam | ab59720; RRID:AB_946249 |
| Mouse anti-NeuN | Abcam | ab104224; RRID:AB_10711040 |
| Mouse anti-TUBB3 | Proteintech | 66375-1-Ig; RRID:AB_2814998 |
| Mouse anti-GAPDH | ZSGB-Bio, Beijing, P R China | TA-08; RRID:AB_2747414 |
| Mouse anti-β-actin | ZSGB-Bio | TA-09; RRID:AB_2636897 |
| Donkey anti-Rabbit IgG, Alexa Fluor 488 | Thermo Fisher Scientific, Waltham, MA, USA | A-21206, RRID:AB_2535792 |
| Donkey anti-Mouse IgG, Alexa Fluor 594 | Thermo Fisher Scientific | A-21203, RRID:AB_141633 |
| **Commercial Assays** | | |
| Stool Genomic DNA Extraction Kit | Solarbio, Beijing, P R China | D2700 |
| UNIQ-10 Column Micro DNA Gel Extraction Kit | Sangon Biotech | B511139 |
| PrimeScript™ RT Reagent kit | TaKaRa, Shiga, Japan | RR037A |
| SYBR® Premix Ex Taq II | TaKaRa | DRR081A |
| Mouse Lipopolysaccharides (LPS) ELISA Kit | CUSABIO, College Park, MS, USA | CSB-E13006m |
| Mouse IL-1β ELISA Kit | Enzyme-linked Biotech, Shanghai, P R China | ml001814 |
| Luminex Multiplex Assays | Bio-Rad, Hercules, CA, USA | LX-MultiDTM-10 |
| Mouse Cytokine Array Kit | R&D Systems, Minneapolis, MN, USA | ARY006 |
| **Chemicals** | | |
| BIOTICS probiotics | Christian D.A. Hansen, Hørsholm, Denmark | N/A |
| HP-β-CD | Sangon Biotech | A600388 |
| Amphotericin B Solution | Sangon Biotech | B540721 |
| Neomycin trisulfate salt hydrate | Sangon Biotech | A610366 |
| Metronidazole | Sangon Biotech | A600633 |
| Ampicillin sodium salt | Sangon Biotech | A100339 |
| Vancomycin hydrochloride | Sangon Biotech | A100990 |
| Hydrocortisone | Sangon Biotech | A610506 |
| PEG4000 | Sangon Biotech | A620431 |
| FITC-dextran mol wt 4000 (FD4) | Sigma-Aldrich, St. Louis, MO, USA | 46944 |
| **Software** | | |
| GraphPad Prism 8 | GraphPad Prism | N/A |
| Image J | NIH | https://imagej.nih.gov/ij/download.html |
| R 4.1.0 | Cran R project | N/A |
| Genescloud tools | Personalbio Technology, Shanghai, P R China | https://www.genescloud.cn |
| Leica LAS X | Leica, Wetzlar, Germany | N/A |
| Zeiss ZEN | Zeiss, Oberkochen, Germany | N/A |
| SMART™ tracking software program | San Diego Instruments, CA, USA | N/A |
| Curve Expert 1.4 | Daniel Hyams, Hixson, TN, USA | N/A |

**Supplementary Table. 2:** Primer sequences used for verification of ABx administration before FMT.

| **Target** | **Primer sequence** |
| --- | --- |
| 16S rRNA gene | 27-F：5’-AGA GTT TGA TCC TGG CTC AG-3’ |
|  | 1541-R：5’-AAG GAG GTG ATC CAG CCG CA-3’ |
| V3-V4 region | 331-F：5’-TCC TAC GGG AGG CAG CAG T-3’ |
|  | 797-R：5’-GGA CTA CCA GGG TAT CTA ATC CTG TT-3’ |

**Supplementary Table. 3:** Primer sequences used for RT-qRCR.

| **Gene** | **Primer sequence** |
| --- | --- |
| Reg3g | Forward: 5’-CAT CCA CCT CTG TTG GGT TC-3’ |
|  | Reverse: 5’-TTC CTG TCC TCC ATG ATC AAA-3’ |
| Lcn2 | Forward: 5’-GAG CTG TCC CCT GAA CTG AA-3’ |
|  | Reverse: 5’-CAG TCA GCC ACA CTC ACC AC-3’ |

**Supplementary Table. 4:** Statistical data.

| FIGURE | METHODS | DF/K-W statistic | P VALUE |
| --- | --- | --- | --- |
| Fig. 1b | Two-way ANOVA | Interaction: F_(39, 392)_=0.5892 | 0.9775 |
|  |  | Row Factor: F_(13, 392)_=18.69 | <0.001 |
|  |  | Column Factor: F_(3, 392)_=63.6 | <0.001 |
| Fig. 1c | Two-way ANOVA | Interaction: F_(36, 156)_=2.393 | <0.001 |
|  |  | Row Factor: F_(12, 156)_=10.71 | <0.001 |
|  |  | Column Factor: F_(3, 156)_=192.2 | <0.001 |
| Fig. 1d | One-way ANOVA | F_(3, 28)_ = 7.036 | 0.0011 |
| Fig. 1e | One-way ANOVA | F_(3, 28)_ = 4.992 | 0.0067 |
| Fig. 1f | One-way ANOVA | F_(3, 28)_ = 13.21 | <0.001 |
| Fig. 1g | One-way ANOVA | F_(3, 28)_ = 6.223 | 0.0023 |
| Fig. 1l | Kruskal-Wallis test | 18.13 (Chao1) | <0.001 |
|  |  | 17.82 (Goods's coverage) | <0.001 |
|  |  | 16.1 (Observed species) | 0.0011 |
|  |  | 11.61 (Shannon) | 0.0089 |
| Fig. 1n | One-way ANOVA | F_(3, 28)_ = 12.11 | <0.001 |
| Fig. 1o | One-way ANOVA | F_(3, 20)_ = 10.9 | <0.001 |
| Fig. 1q | One-way ANOVA | F_(3, 28)_ = 5.797 (GluA1) | 0.0033 |
|  |  | F_(3, 28)_ = 0.3393 (GluA2) | 0.797 |
|  |  | F_(3, 28)_ = 6.542 (PSD-95) | 0.0017 |
|  |  | F_(3, 28)_ = 3.75 (SYP) | 0.0221 |
| Fig. 2b | Unpaired t test | t = 3.002 | 0.0095 |
| Fig. 2c | Unpaired t test | t = 2.689 | 0.0176 |
| Fig. 2d | Unpaired t test | t = 3.001 | 0.0095 |
| Fig. 2e | Unpaired t test | t = 4.25 | <0.001 |
| Fig. 2f | Unpaired t test | t = 3.249 | 0.0058 |
| Fig. 2g | Unpaired t test | t = 3.952 | 0.0014 |
| Fig. 2h | Unpaired t test | t = 2.795 (LPS) | 0.0143 |
|  |  | t = 3.924 (IL-1β) | 0.0015 |
| Fig. 2k | Mann-Whitney test | U = 13 (Chao1) | 0.0499 |
|  |  | U = 10 (Observed species) | 0.0207 |
|  |  | U = 8 (Pielou's evenness) | 0.0104 |
|  |  | U = 11 (Shannon) | 0.0281 |
|  |  | U = 12 (Simpson) | 0.0379 |
| Fig. 2n | Unpaired t test | t = 5.092 (GluA1) | <0.001 |
|  |  | t = 0.1986 (GluA2) | 0.8455 |
|  |  | t = 2.318 (PSD-95) | 0.0361 |
|  |  | t = 0.8110 (SYP) | 0.4309 |
| Fig. 2p | Unpaired t test | t = 2.291 (TrkB) | 0.038 |
|  |  | t = 1.866 (ERK1/2) | 0.0831 |
|  |  | t = 0.2474 (NGF) | 0.8082 |
|  |  | t = 4.105 (BDNF) | 0.0011 |
| Fig. 2s | Unpaired t test | t = 3.665 | 0.0014 |
| Fig. 2t | Unpaired t test | t = 4.11 (NLRP3) | 0.0011 |
|  |  | t = 2.54 (ASC) | 0.0236 |
|  |  | t = 3.561 (NF-κB) | 0.0031 |
| Fig. 3b | Two-way ANOVA | Interaction: F_(36, 416)_=2.393 | 0.9943 |
|  |  | Row Factor: F_(12, 416)_=10.71 | <0.001 |
|  |  | Column Factor: F_(3, 416)_=192.2 | <0.001 |
| Fig. 3c | Two-way ANOVA | Interaction: F_(36, 156)_=2.393 | <0.001 |
|  |  | Row Factor: F_(12, 156)_=10.71 | <0.001 |
|  |  | Column Factor: F_(3, 156)_=192.2 | <0.001 |
| Fig. 3e | One-way ANOVA | F_(3, 32)_ = 6.115 | 0.0021 |
| Fig. 3f | One-way ANOVA | F_(3, 32)_ = 4.417 | 0.0104 |
| Fig. 3g | One-way ANOVA | F_(3, 32)_ = 9.531 | <0.001 |
| Fig. 3l | Kruskal-Wallis test | 15.68 (Chao1) | 0.0013 |
|  |  | 16.72 (Observed species) | <0.001 |
|  |  | 18.26 (Shannon) | <0.001 |
|  |  | 8.243 (Faith's PD) | 0.0412 |
|  |  | 15.78 (Pielou's evenness) | 0.0013 |
|  |  | 14.96 (Goods's coverage) | 0.0018 |
| Fig. 3m | One-way ANOVA | F_(3, 32)_ = 8.951 (LPS) | <0.001 |
|  |  | F_(3, 32)_ = 5.436 (IL-1β) | 0.0039 |
|  |  | F_(3, 32)_ = 14.2 (TNF-α) | <0.001 |
|  |  | F_(3, 32)_ = 6.221 (IFN-γ) | 0.0019 |
|  |  | F_(3, 32)_ = 4.808 (IL-6) | 0.0071 |
|  |  | F_(3, 32)_ = 4.919 (IL-4) | 0.0064 |
|  |  | F_(3, 32)_ = 2.570 (IL-10) | 0.0715 |
|  |  | F_(3, 32)_ = 5.64 (IL-12p70) | 0.0032 |
| Fig. 3n | One-way ANOVA | F_(3, 32)_ = 25.18 | <0.001 |
| Fig. 3o-1 | One-way ANOVA | F_(3, 32)_ = 7.854 (GluA1) | <0.001 |
|  |  | F_(3, 32)_ = 0.6754 (GluA2) | 0.5744 |
|  |  | F_(3, 32)_ = 7.312 (PSD-95) | <0.001 |
|  |  | F_(3, 32)_ = 9.161 (SYP) | <0.001 |
| Fig. 3o-2 | One-way ANOVA | F_(3, 32)_ = 6.64 (TrkB) | 0.0016 |
|  |  | F_(3, 32)_ = 9.913 (ERK1/2) | <0.001 |
|  |  | F_(3, 32)_ = 15.31 (NGF) | <0.001 |
|  |  | F_(3, 32)_ = 7.318 (BDNF) | <0.001 |
| Fig. 4b | Unpaired t test | t = 2.532 | 0.0209 |
| Fig. 4c | Unpaired t test | t = 2.765 | 0.0128 |
| Fig. 4d | Unpaired t test | t = 2.838 | 0.0109 |
| Fig. 4e | Unpaired t test | t = 2.147 | 0.0456 |
| Fig. 4f | Unpaired t test | t = 3.428 | 0.003 |
| Fig. 4k | Mann-Whitney test | U = 36 (Chao1) | 0.3150 |
|  |  | U = 34 (Observed species) | 0.2475 |
|  |  | U = 12 (Shannon) | 0.0029 |
|  |  | U = 3 (Simpson) | <0.001 |
|  |  | U = 7 (Pielou’s evenness) | <0.001 |
| Fig. 4n | Unpaired t test | t = 2.949 (ZO-1) | 0.0106 |
|  |  | t = 3.656 (Occludin) | 0.0026 |
|  |  | t = 2.603 (Claudin-5) | 0.0208 |
| Fig. 4o | Unpaired t test | t = 2.927 | 0.0099 |
| Fig. 5a | Unpaired t test | t = 3.548 (LPS) | 0.0023 |
|  |  | t = 2.342 (IL-1β) | 0.0309 |
|  |  | t = 4.651 (TNF-α) | <0.001 |
|  |  | t = 4.361 (IFN-γ) | <0.001 |
|  |  | t = 2.258 (IL-6) | 0.0366 |
|  |  | t = 3.423 (IL-4) | 0.003 |
|  |  | t = 3.035 (IL-10) | 0.0071 |
|  |  | t = 3.276 (IL-12p70) | 0.0042 |
| Fig. 5b | Unpaired t test | t = 4.525 (Iba1) | <0.001 |
|  |  | t = 2.243 (GFAP) | 0.0416 |
| Fig. 5c | Unpaired t test | t = 3.085 (Microglia) | 0.0054 |
|  |  | t = 5.443 (Astrocyte) | <0.001 |
| Fig. 5f | Unpaired t test | t = 12.08 (NLRP3) | <0.001 |
|  |  | t = 2.334 (Caspase-1) | 0.03 |
|  |  | t = 6.15 (ASC) | <0.001 |
|  |  | t = 2.98 (IL-18) | 0.0099 |
|  |  | t = 3.414 (NF-κB) | 0.0042 |
| Fig. 5h | Unpaired t test | t = 4.796 (GluA1) | <0.001 |
|  |  | t = 1.142 (GluA2) | 0.2727 |
|  |  | t = 2.386 (PSD-95) | 0.0317 |
|  |  | t = 0.5247 (SYP) | 0.608 |

**Supplemental figures**

**Supplementary Fig. 1:** Blood ethanol concentration, supplementary data of behavioral tests, and representative cortisol LC-MS chromatograms. *Related to* ***Fig. 1.***

(a) Blood ethanol concentration of mice. (b) Distance travelled in OFT. (c) Representative tracks in OFT. (d) Representative tracks in EPM. (e) Representative LC-MS chromatogram of cortisol.


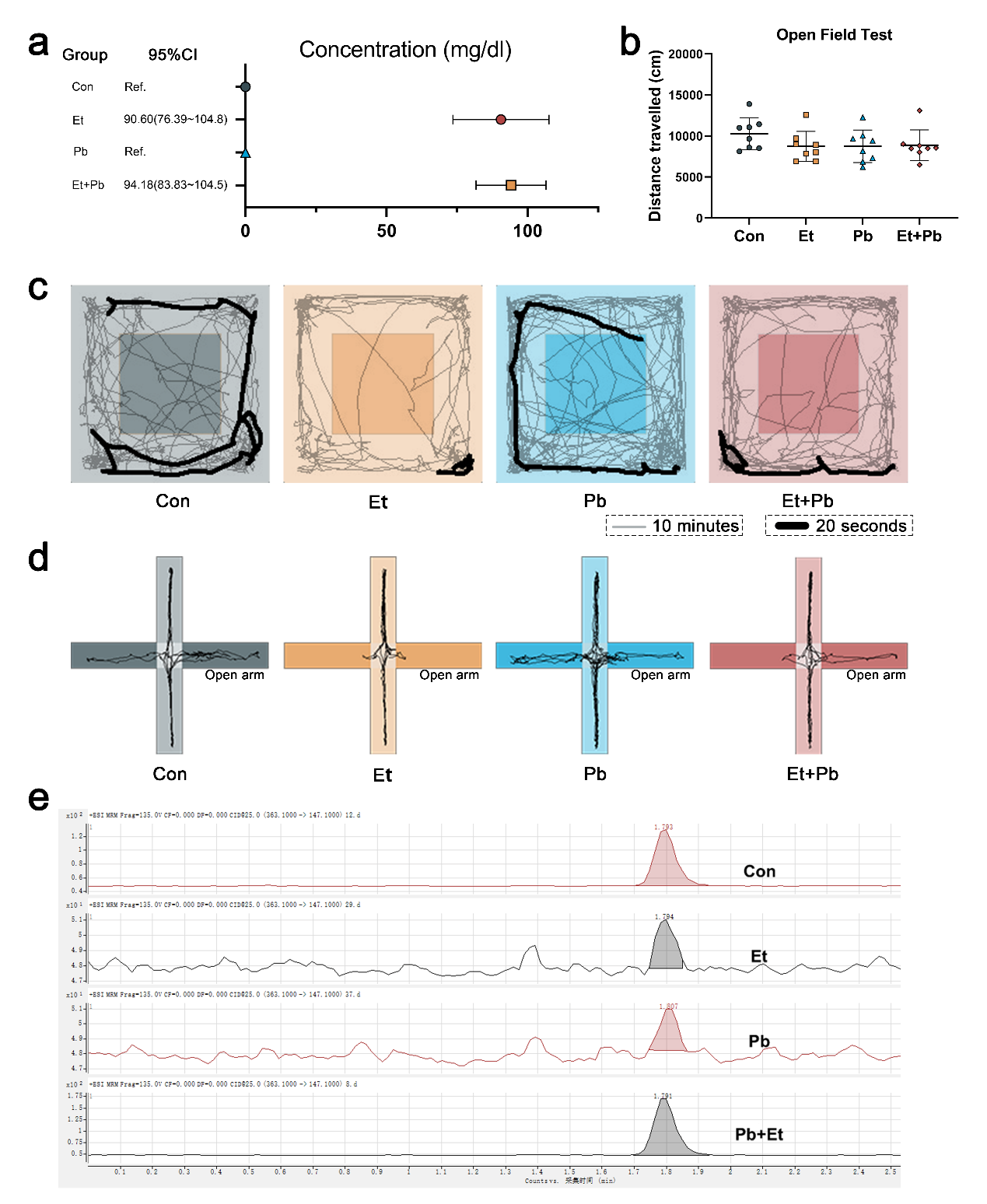


**Supplementary Fig. 2:** Supplementary data of 16S rRNA gene sequencing. *Related to* ***Fig. 1.***

(a) Venn diagram of the OTUs. (b) The legend of Fig. 1j. (c) Supplemental data of α-diversity related to Fig. 1l. (d) Heatmap shows the distribution trends of species abundance in each sample.


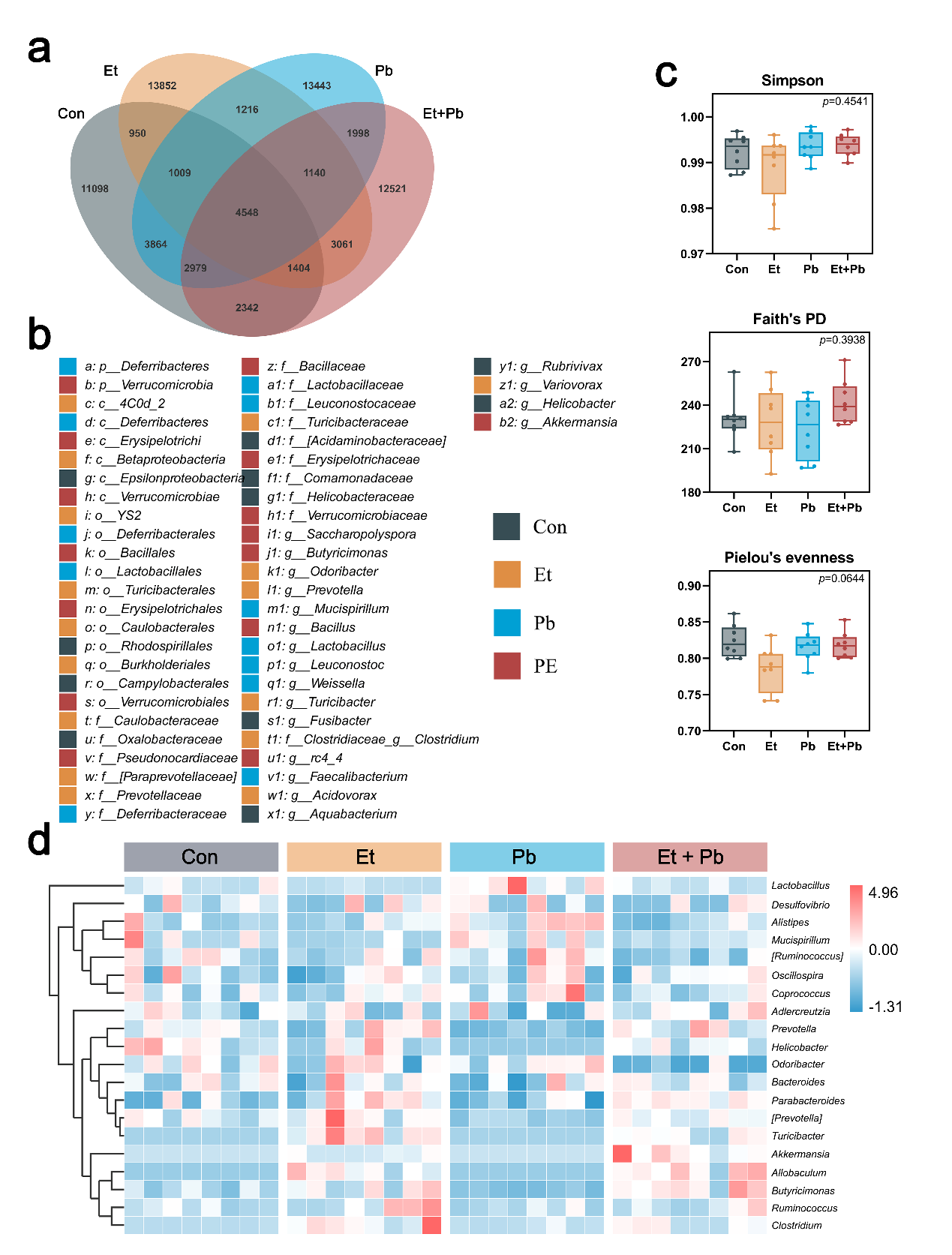


**Supplementary Fig. 3:** Supplementary data about ileum, serum and hippocampus. *Related to* ***Fig. 1.***

(a) RT-qPCR detected the Lcn2 and Reg3g mRNA levels in ileum tissue. (b) Serum LPS and IL-1β levels and the correlation with the immobility in FST. (c) Representative Nissl staining of hippocampal CA3 and CA1 regions.


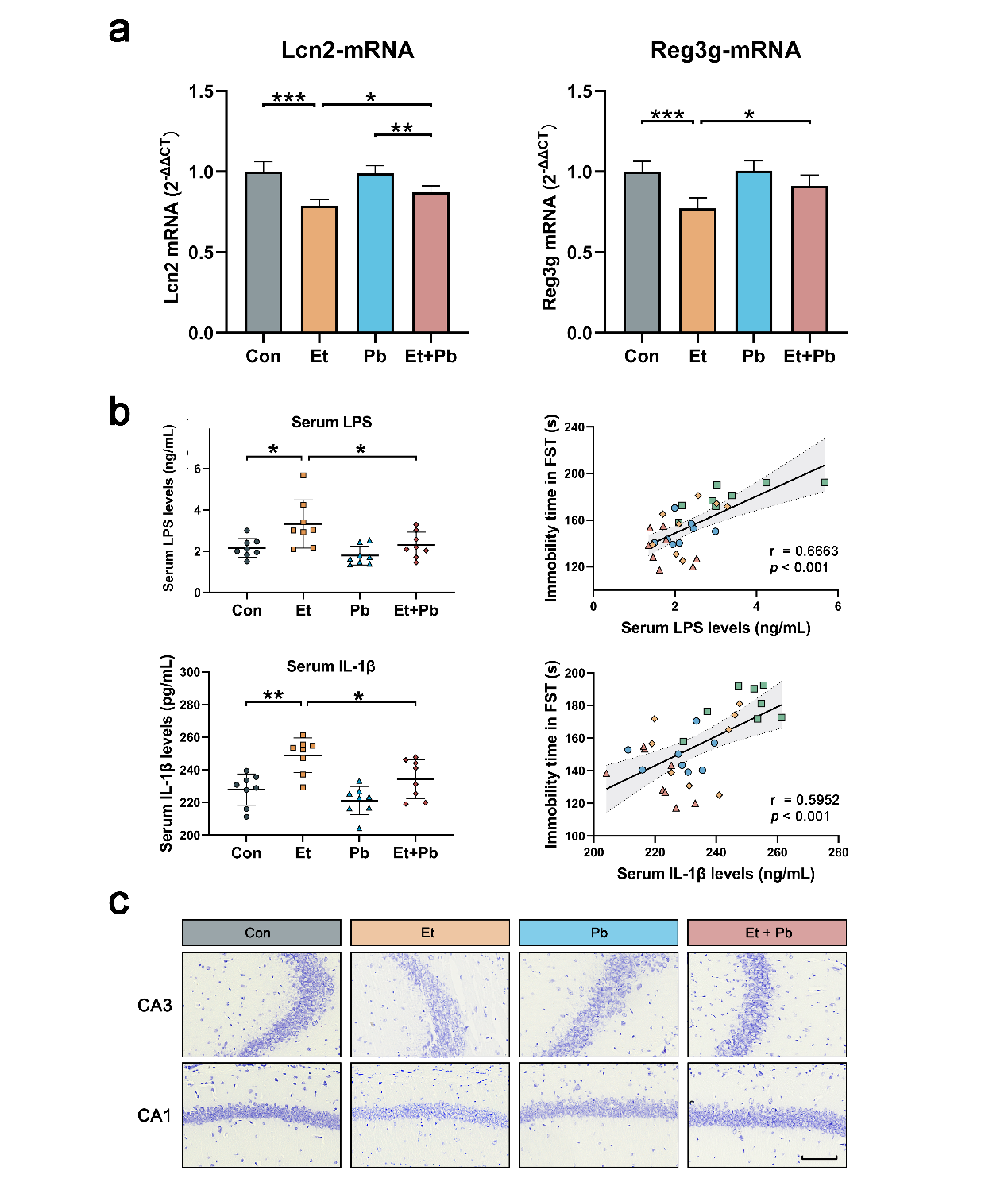


**Supplementary Fig. 4:** Preparation for FMT: cleaning of gut microbiota and examination of filtrate for transplantation. *Related to* ***Fig. 2.***

(a) Verification process of cleaning effect. (b) DNA was isolated from the stool of mice, and 16S rRNA gene was measured by qPCR. (c) The PCR products from **c** were run on an agarose gel for a general comparison of the four groups. (d) Stools were resuspended in thioglycolate and plated on non-selective agar to measure gut bacterial load. (e) Ethanol content in filtrate for transplantation was determined by HS-GC.


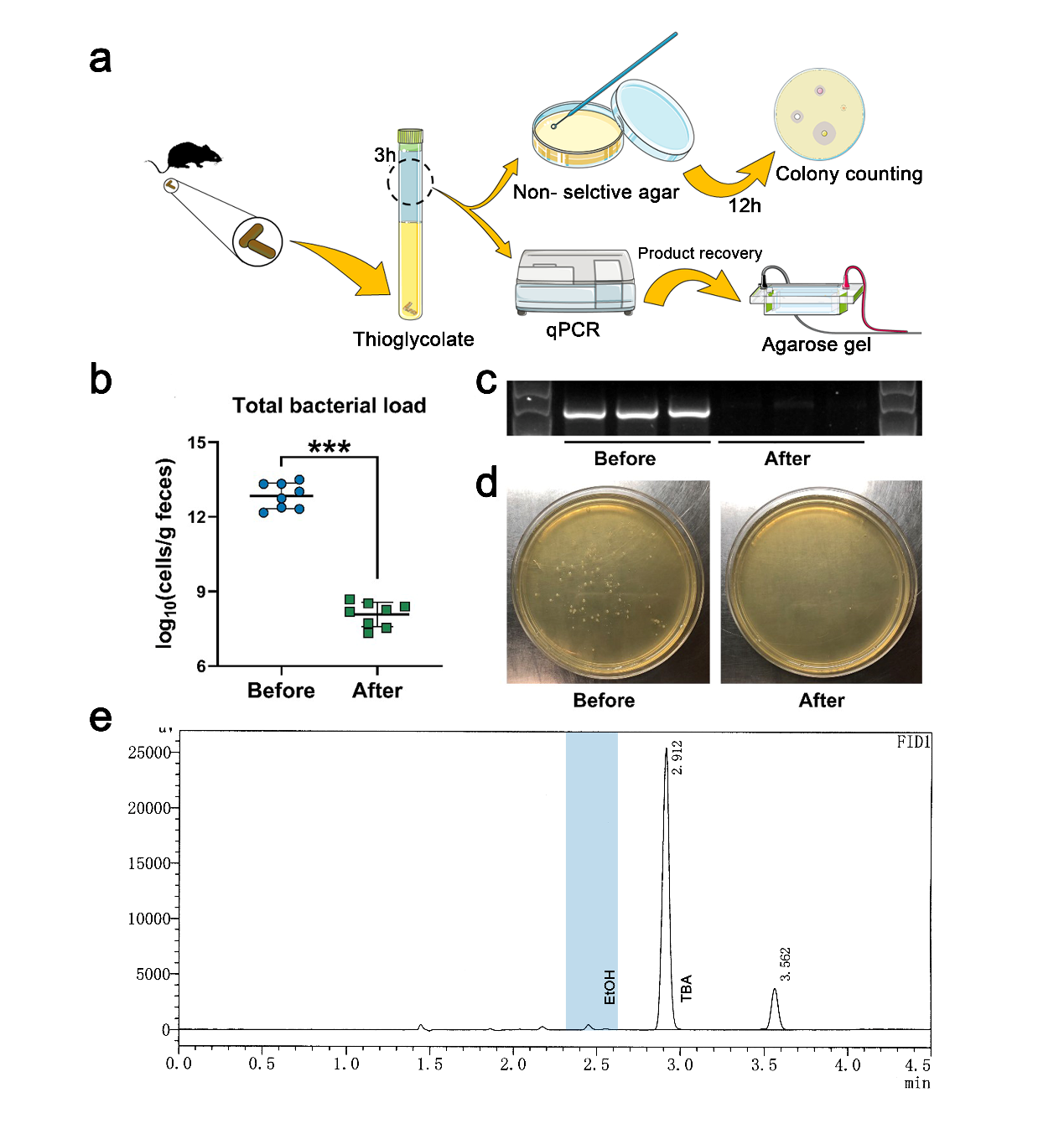


**Supplementary Fig. 5:** Supplementary data of behavioral tests, and representative cortisol LC-MS chromatograms. *Related to* ***Fig. 2.***

(a) Distance travelled in OFT. (b) Total liquid consumption in SPT. (c) Sucrose consumption in SPT. (d) Representative tracks in OFT. (e) Representative tracks in EPM. (f) Representative LC-MS chromatogram of cortisol.


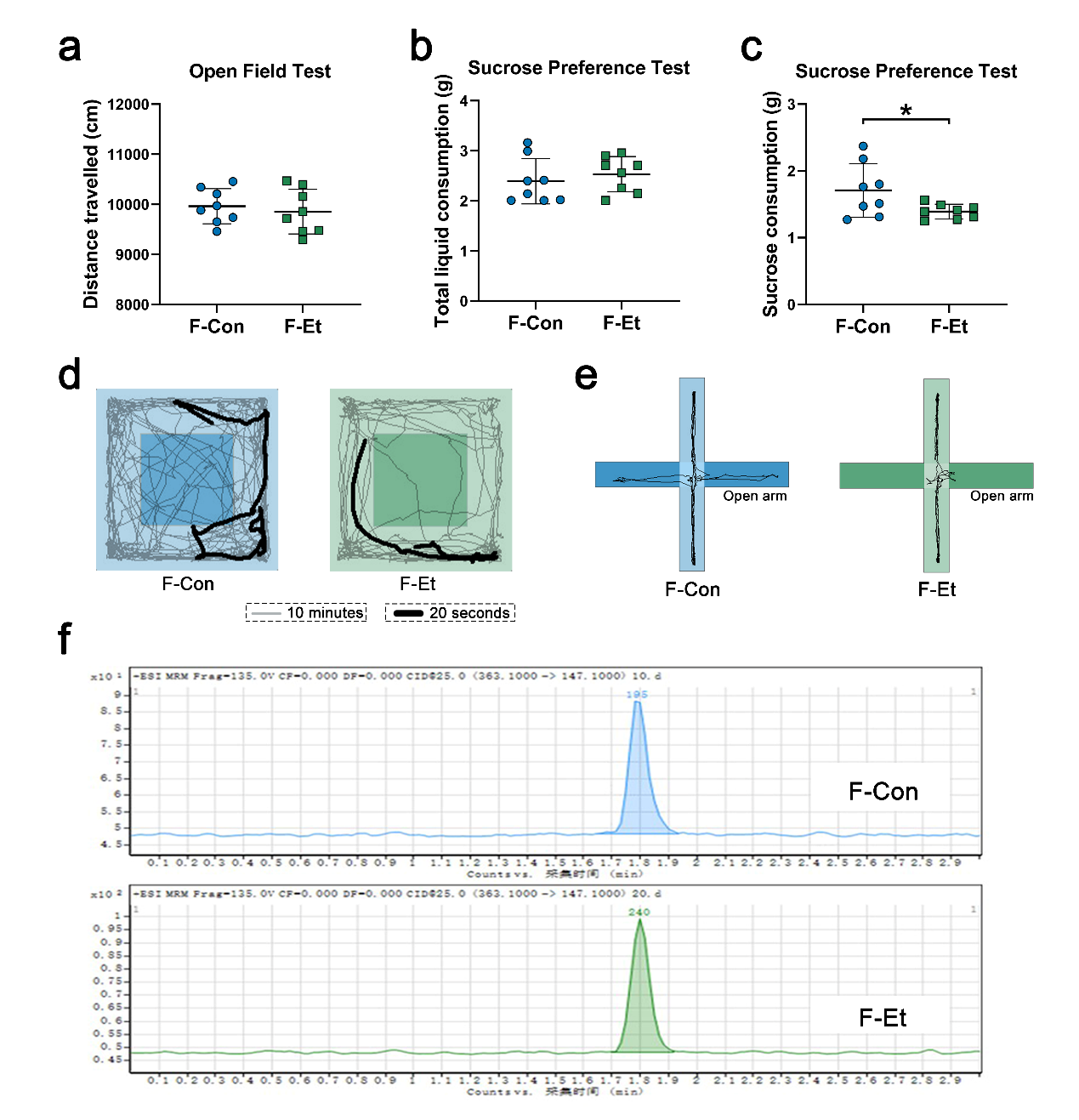


**Supplementary Fig. 6:** Supplementary data of 16S rRNA gene sequencing. *Related to* ***Fig. 2.***

(a) Supplemental data of α-diversity related to Fig. 2k. (b) Taxonomic differences are based on 16S rRNA gene sequences extracted from the metagenome. (c) LEfSe analysis showed the significantly enriched microbiome in each group.


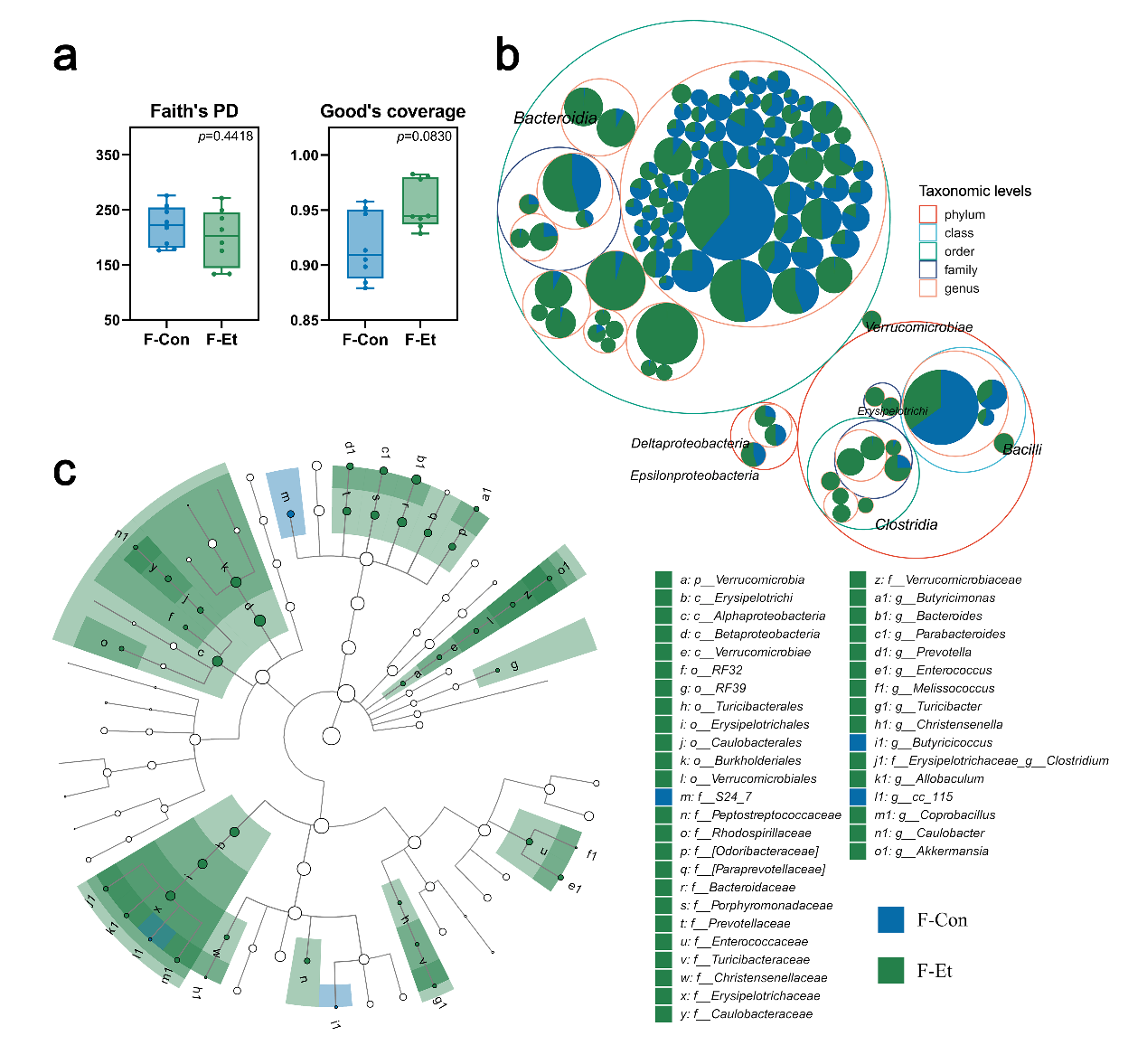


**Supplementary Fig. 7:** The legend of mouse cytokine array. *Related to* ***Fig. 2u.***

*: Significantly increased in F-Et group, *P* < 0.05.


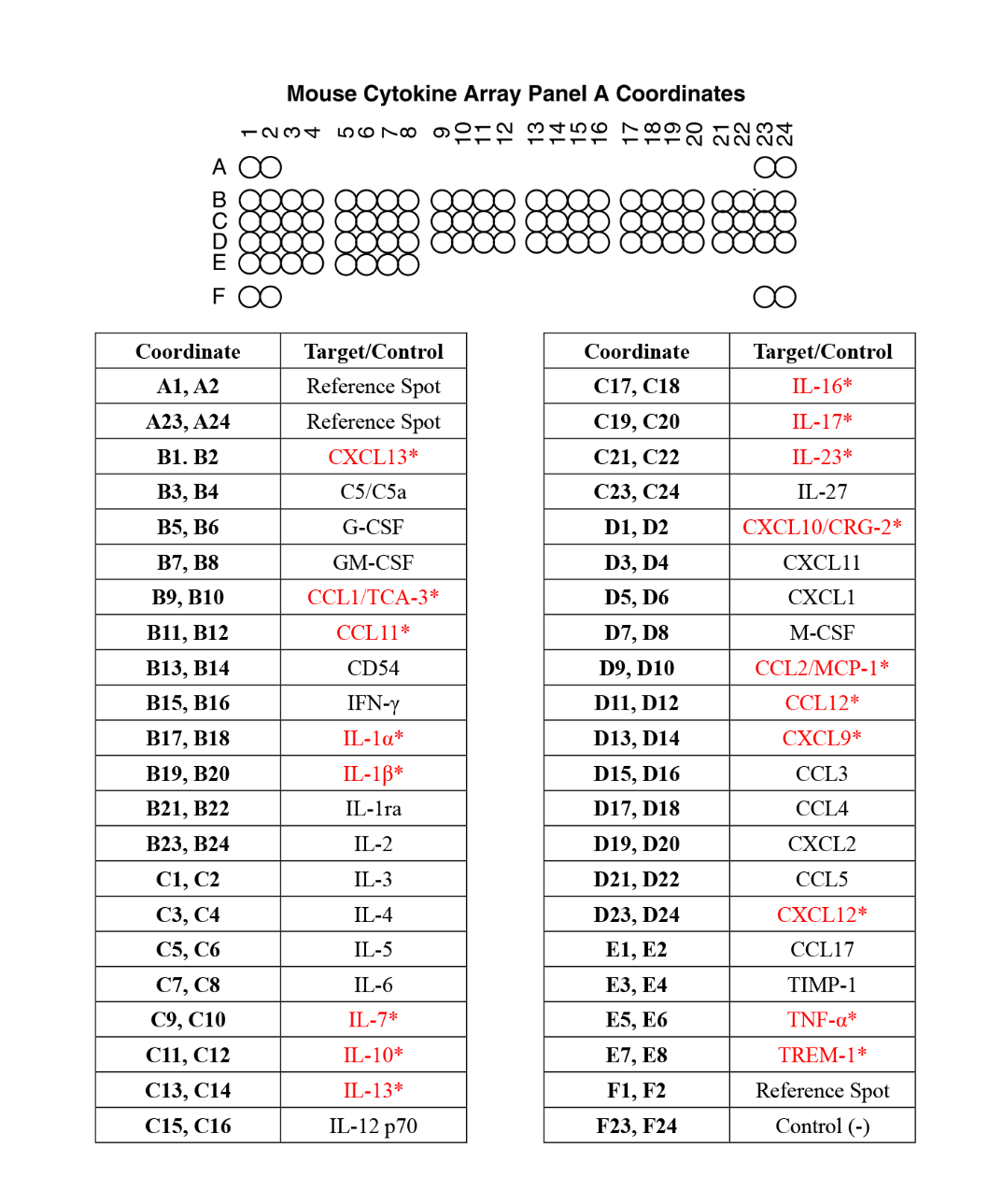


**Supplementary Fig. 8:** AAV transfection had no effect on expression of NLRP3 in ileum tissue. *Related to* ***Fig. 3d.***


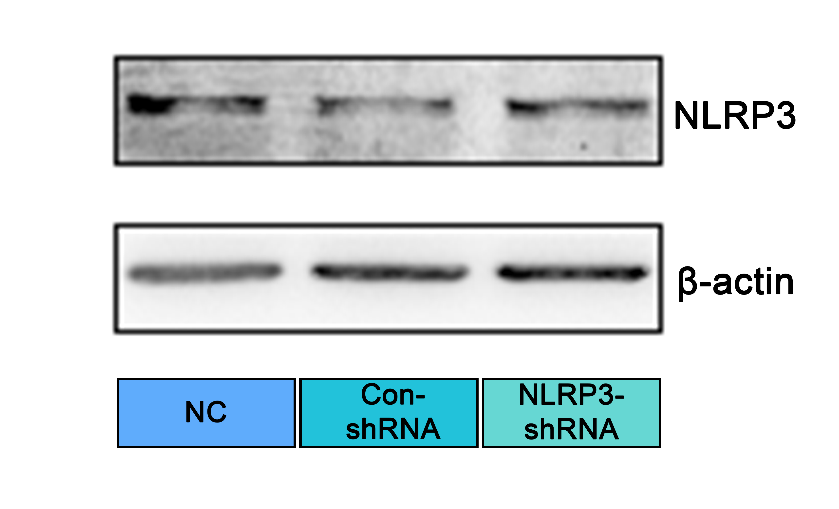


**Supplementary Fig. 9:** Supplementary data of behavioral tests. *Related to* ***Fig. 3.***

(a) Representative tracks in OFT. (b) Distance travelled in OFT. (c) Representative tracks in EPM.


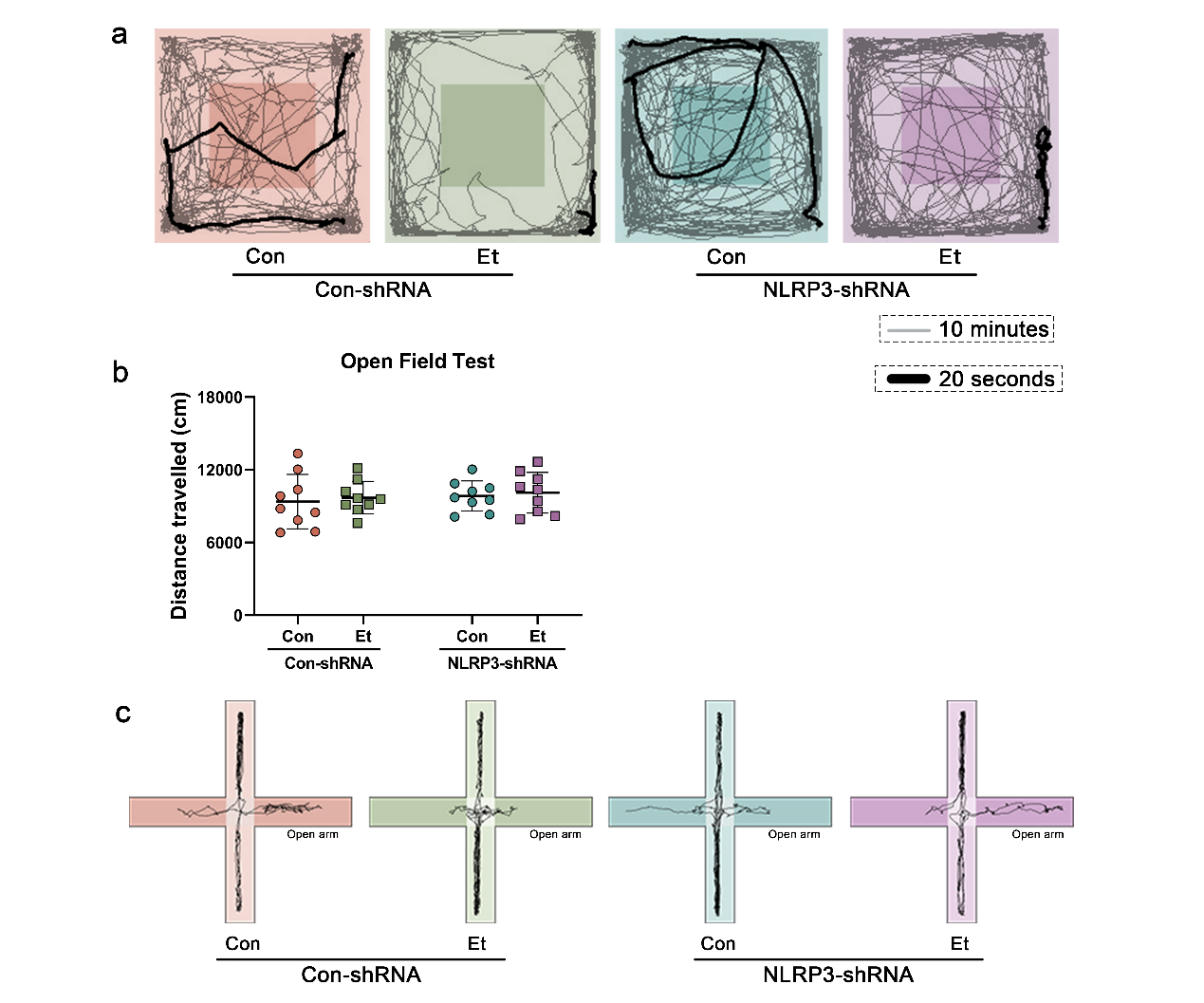


**Supplementary Fig. 10:** Supplementary data of 16S rRNA gene sequencing. *Related to* ***Fig. 3.***

(a) Supplemental data of α-diversity related to Fig. 2I. (b) LEfSe analysis showed the significantly enriched microbiome in each group. (c) Taxonomic differences are based on 16S rRNA gene sequences extracted from the metagenome. (d) Relative abundance of gut microbiota in family level. (e) Relative abundance of gut microbiota in genus level.


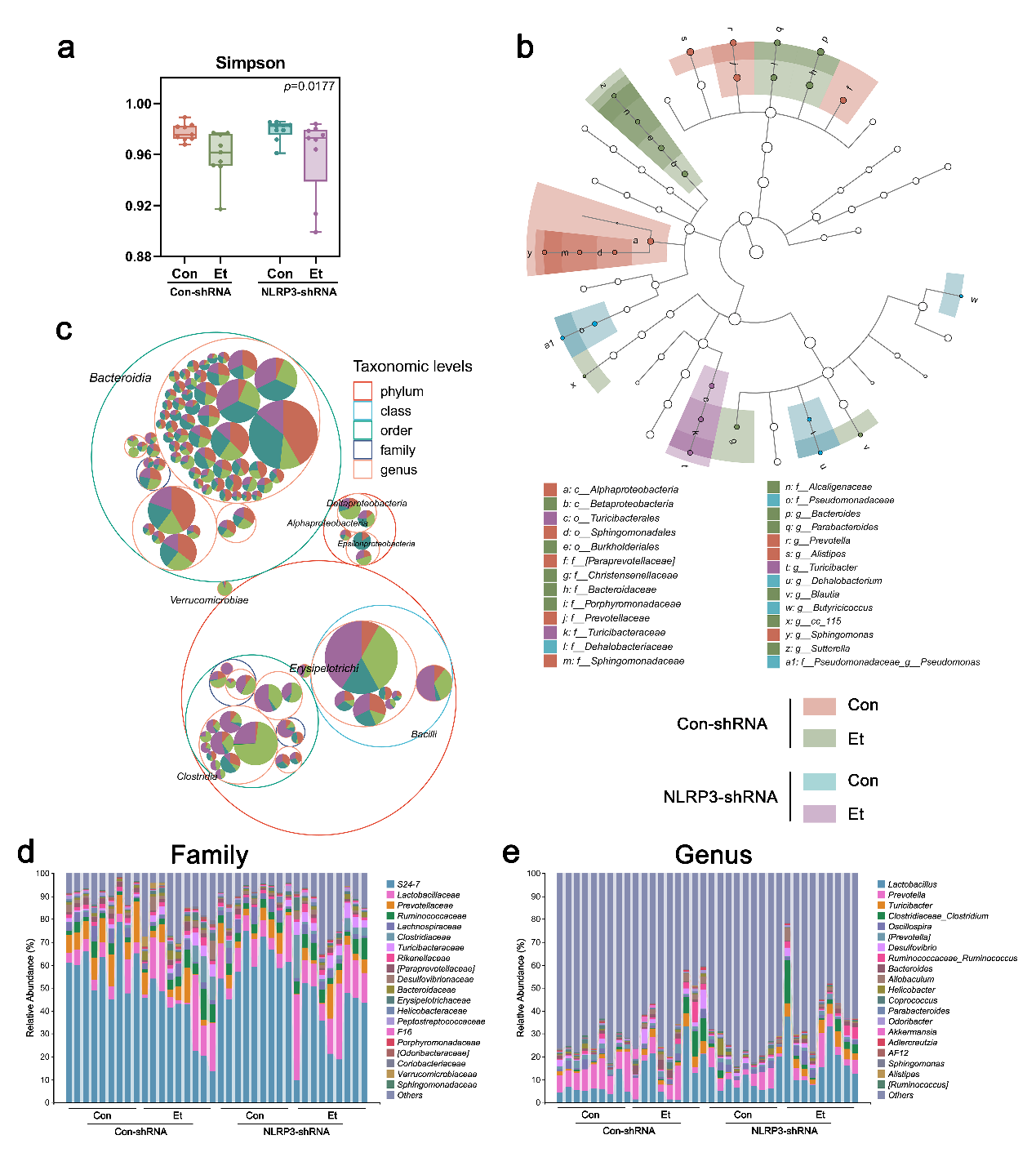


**Supplementary Fig. 11:** Expression of tight junction proteins in ileum tissue, supplemental data of serum inflammatory cytokines. *Related to* ***Fig. 3.***

(a) Western bolt showing expression of tight junction proteins. (b) supplemental data of serum inflammatory cytokines detected by Luminex.


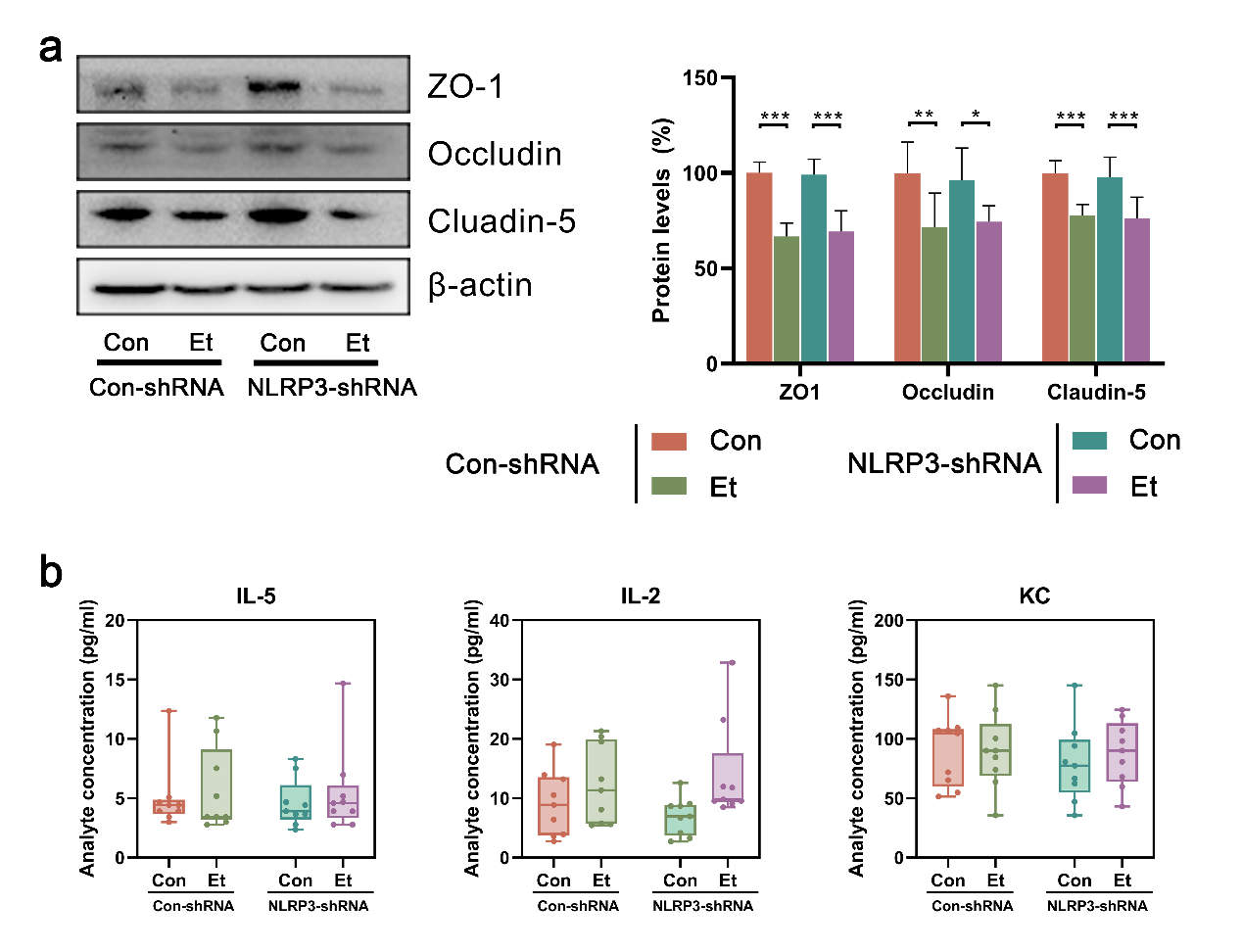


**Supplementary Fig. 12:** Supplementary data of behavior tests and 16S rRNA gene sequencing. *Related to* ***Fig. 4.***

(a) Distance travelled in OFT. (b) Total liquid consumption in SPT. (c) Sucrose consumption in SPT. (d) Venn diagram of the OTUs. (e) Taxonomic differences are based on 16S rRNA gene sequences extracted from the metagenome. (f) LEfSe analysis showed the significantly enriched microbiome in recipient mice. (g) Supplemental data of α-diversity related to Fig. 4k.


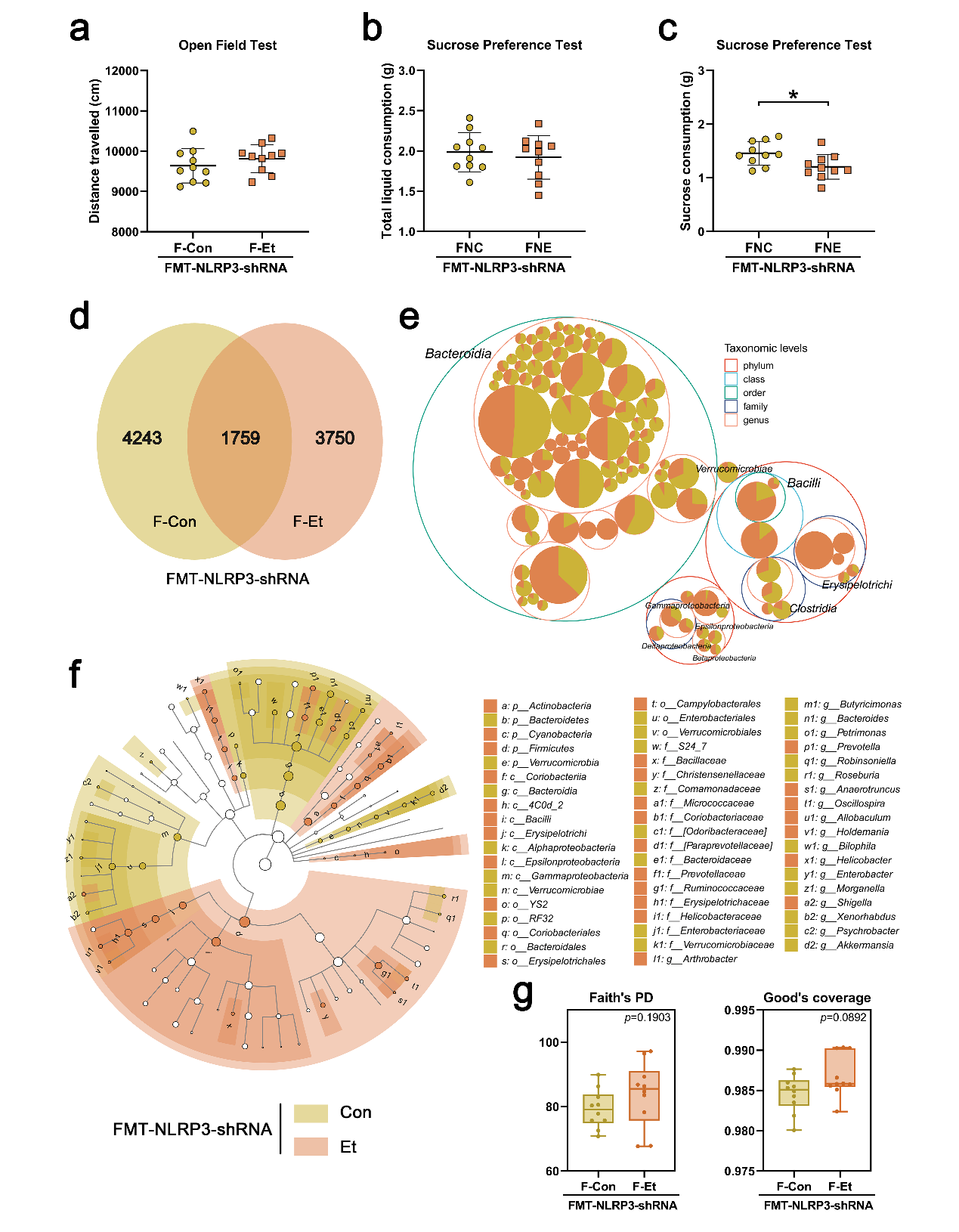


**Supplementary Fig. 13:** Supplementary data of serum inflammatory cytokines detected by Luminex. *Related to* ***Fig. 5a.***


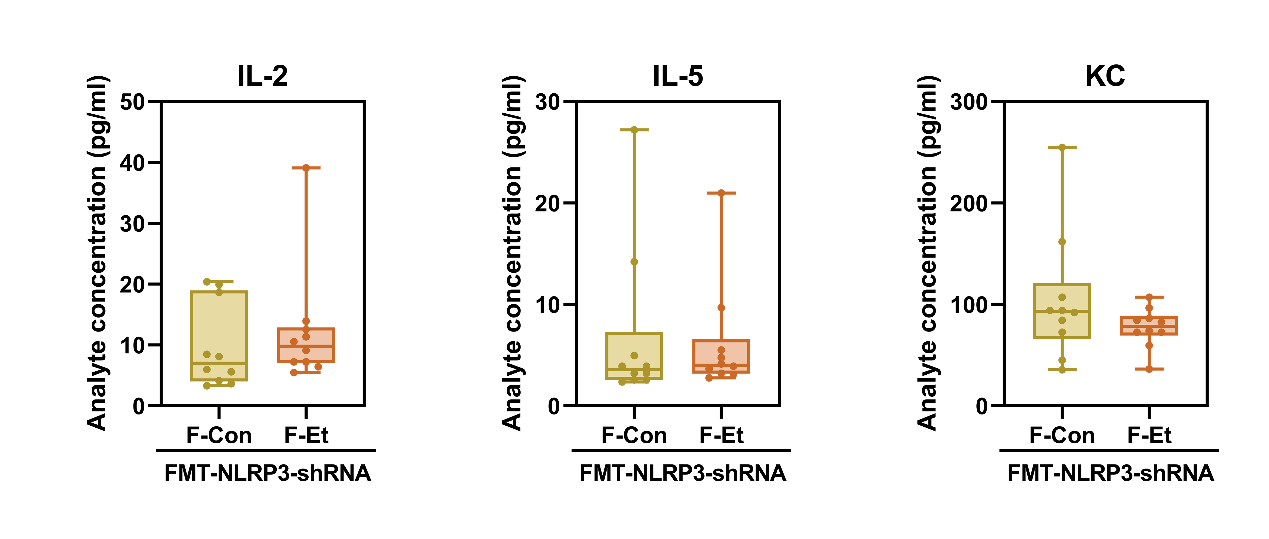


**Supplementary Fig. 14:** FMT from hippocampal NLRP3 down-regulated mice reduced expression of hippocampal neurotrophic proteins in recipient mice. *Related to* ***Fig. 5.***


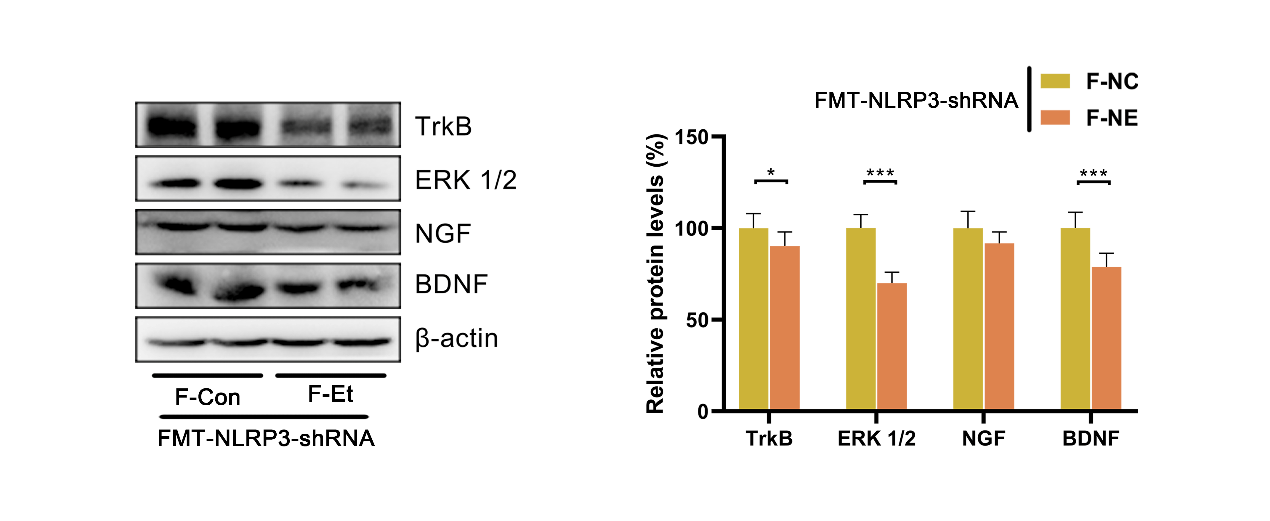


**Supplementary Fig. 15:** Effects of subdiaphragmatic vagotomy (SDV) on behavior of recipient mice.

(a) Experimental design for SDV and FMT from Et groups. (b) Distance travelled in OFT. (c) Time in the central area of the OFT. (d) Time spent in the open arms of the EPM. (e) Immobility time in the FST. (f) Representative tracks in OFT. (g) Representative tracks in EPM.

Data are expressed as the mean ± SD, n=10, **P*<0.05.

**
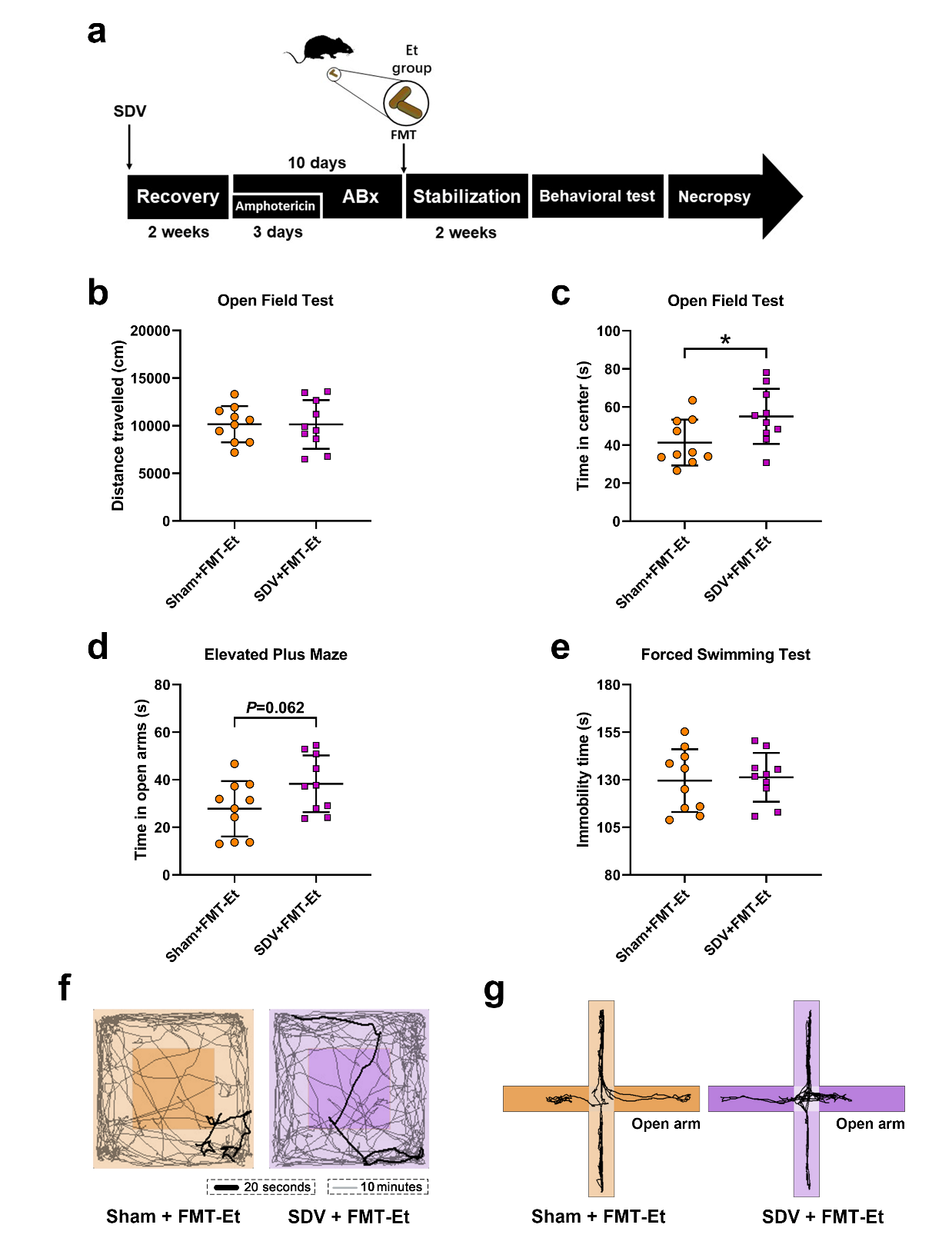
**

**Supplementary Fig. 16:** The mediation model used in Fig. 6c. *Related to* ***Fig. 6c.***

**
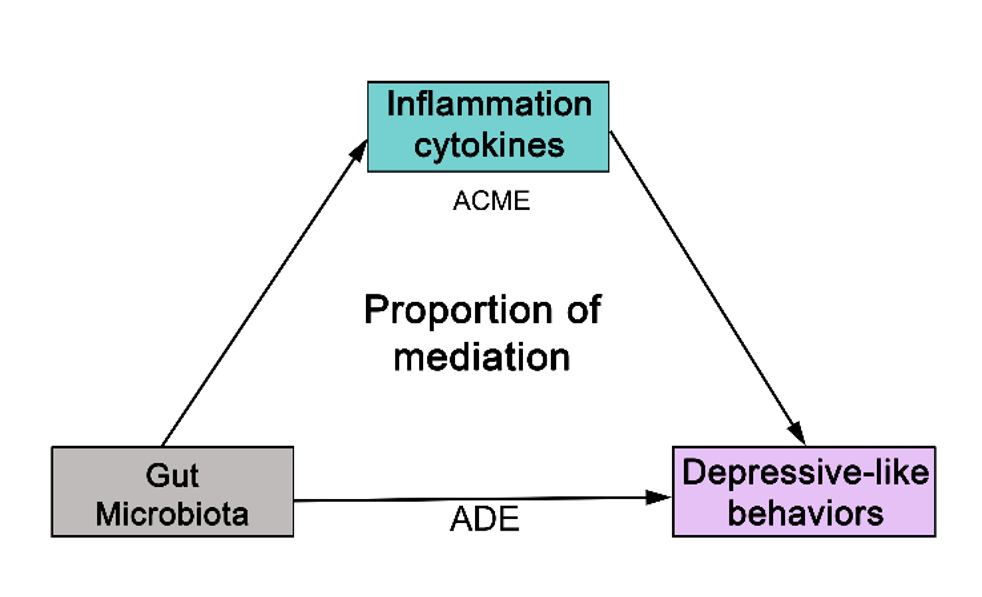
**

**Supplementary Fig. 17:** Graphic abstract

**
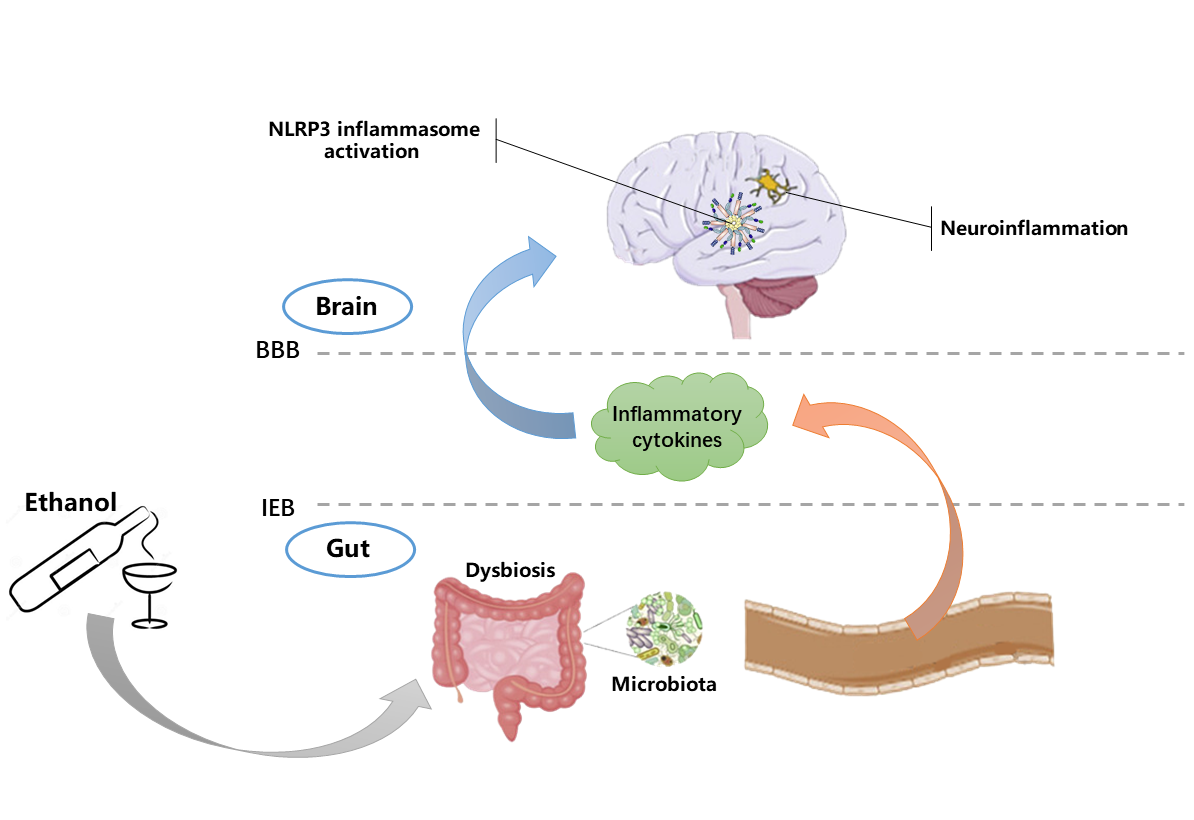
**

**Supplementary Fig. 18:** Raw images of western blots from all Figures (Protein Ladder 26616 produced by Thermo Fisher Scientific).


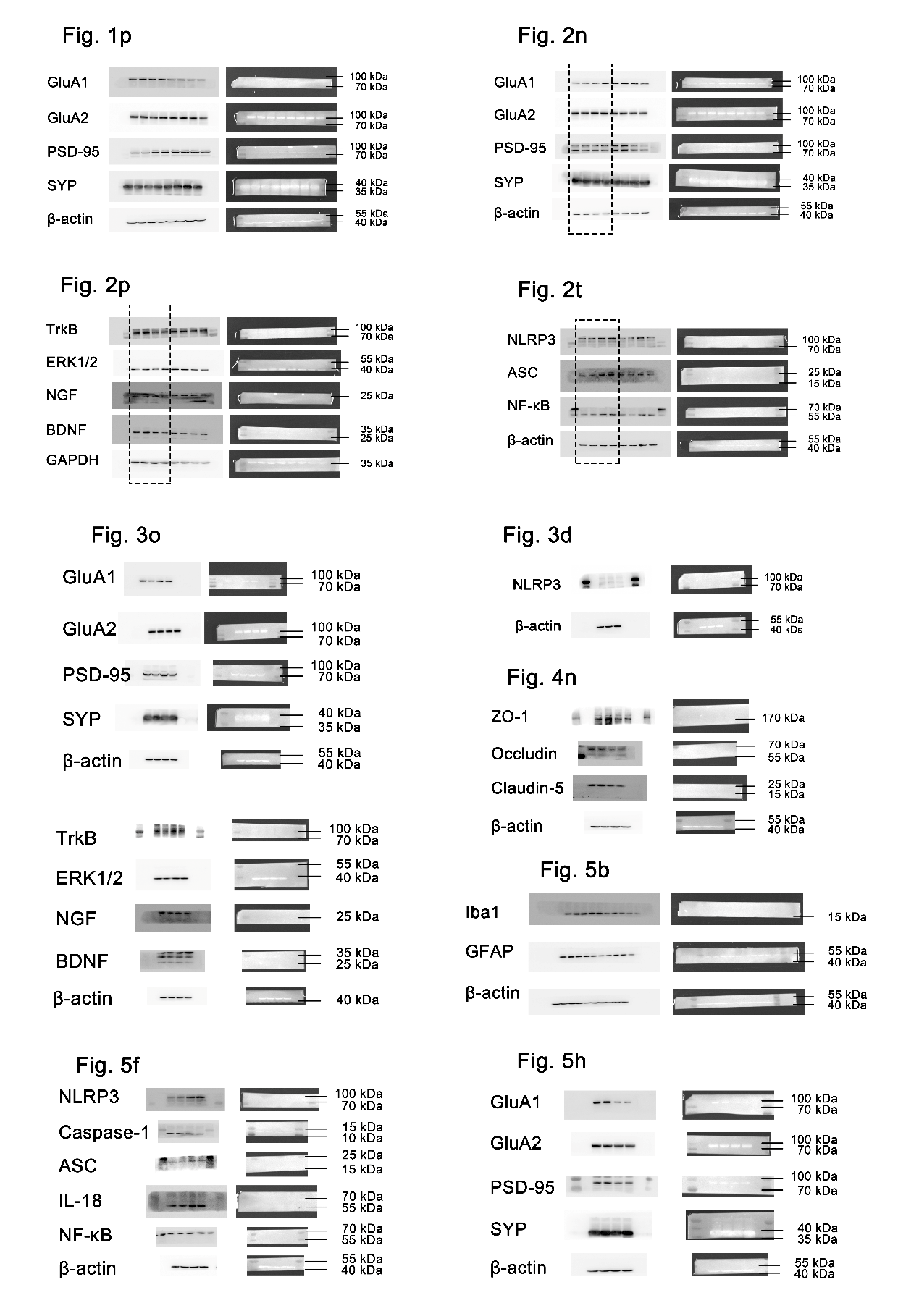

Supplement: Supplementary file 1 — Supplemental information [file 41380_2022_1841_MOESM1_ESM.docx]
